# Comprehensive analysis of the *GALACTINOL SYNTHASE* (*GolS*) gene family in citrus and the function of *CsGolS6* in stress tolerance

**DOI:** 10.1371/journal.pone.0274791

**Published:** 2022-09-16

**Authors:** Cristina P. S. Martins, Denise Fernandes, Valéria M. Guimarães, Dongliang Du, Delmira C. Silva, Alex-Alan F. Almeida, Frederick G. Gmitter, Wagner C. Otoni, Marcio G. C. Costa

**Affiliations:** 1 Centro de Biotecnologia e Genética, Departamento de Ciências Biológicas, Universidade Estadual de Santa Cruz, Ilhéus, Bahia, Brazil; 2 Departamento de Biologia Vegetal, Universidade Federal de Viçosa, Viçosa, Minas Gerais, Brazil; 3 Departamento de Bioquímica e Biologia Molecular, Universidade Federal de Viçosa, Viçosa, Minas Gerais, Brazil; 4 Horticultural Sciences Department, Citrus Research and Education Center, University of Florida, Lake Alfred, Florida, United States of America; United Arab Emirates University, UNITED ARAB EMIRATES

## Abstract

Galactinol synthase (GolS) catalyzes the first and rate-limiting step in the synthesis of raffinose family of oligosaccharides (RFOs), which serve as storage and transport sugars, signal transducers, compatible solutes and antioxidants in higher plants. The present work aimed to assess the potential functions of citrus GolS in mechanisms of stress response and tolerance. By homology searches, eight *GolS* genes were found in the genomes of *Citrus sinensis* and *C*. *clementina*. Phylogenetic analysis showed that there is a *GolS* ortholog in *C*. *clementina* for each *C*. *sinensis GolS*, which have evolved differently from those of *Arabidopsis thaliana*. Transcriptional analysis indicated that most *C*. *sinensis GolS* (*CsGolS*) genes show a low-level tissue-specific and stress-inducible expression in response to drought and salt stress treatments, as well as to ‘*Candidatus* Liberibacter asiaticus’ infection. *CsGolS6* overexpression resulted in improved tobacco tolerance to drought and salt stresses, contributing to an increased mesophyll cell expansion, photosynthesis and plant growth. Primary metabolite profiling revealed no significant changes in endogenous galactinol, but different extents of reduction of raffinose in the transgenic plants. On the other hand, a significant increase in the levels of metabolites with antioxidant properties, such as ascorbate, dehydroascorbate, alfa-tocopherol and spermidine, was observed in the transgenic plants. These results bring evidence that *CsGolS6* is a potential candidate for improving stress tolerance in citrus and other plants.

## Introduction

Galactinol synthase (GolS, EC 2.4.1.123) is an enzyme catalyzing the first, rate-limiting reaction in the raffinose family of oligosaccharides (RFOs) biosynthetic pathway in flowering plants [1]. GolS belongs to the family 8 (GT8) of glycosyltransferases, a major family of enzymes responsible for the synthesis of diverse sugar conjugates important in structure, storage, energy and signaling [2]. GolS is the primary bottleneck in RFO flux, synthesizing galactinol using UDP-galactose and L-*myo*-inositol as substrates [1]. Galactinol is then used as building blocks in the synthesis of raffinose, stachyose and other RFOs. In addition, GolS acts as an interconnection point between RFO biosynthesis and the central metabolism of inositol, as well as regulates inositol entrance into the process [1].

RFOs play a wide range of predicted functions in plants. These include protection of embryos from maturation-associated seed desiccation [3], carbon storage and transport [4], signal transduction [5–7], osmoprotection of vegetative tissues in response to a range of environmental stresses [1, 8, 9], signaling molecule following pathogen attack and wounding [10, 11], and protection of cellular metabolism from oxidative damage caused by stress conditions due to their intrinsic antioxidant properties [12, 13]. Raffinose was also reported to exist in the chloroplast [14] and evidenced to play a role in stabilizing photosystem II [15].

GolS enzymes are typically encoded by several genes that show developmental and spatial regulation. In *Arabidopsis thaliana*, there are seven *GolS* genes, of which *AtGolS1* and *AtGolS2* are highly expressed in mature and dry seeds, and the other *AtGolS* genes are expressed at low levels in seeds [16]. *AtGolS1* is induced by heat [17], salt and drought stress, whereas *AtGolS2* is induced only by drought and salt stresses and *AtGolS3* only by cold stress [16]. In *Ajuga reptans*, there are two cold-inducible *GolS* genes whose transcription is spatially regulated, which one contributing to the synthesis of a different pool of RFOs, whether storage or transport pool [18]. In cucumber, there are four stress-inducible *GolS* genes, of which *CsGolS1* is expressed in vascular tissues, whereas the other three *CsGolS* genes are expressed in mesophyll cells [4]. In rice, the two *GolS* gene isoforms, *OsGolS1* and *OsGolS2*, were shown to be regulated post-transcriptionally through intron retention in response to abiotic stress and associated with the regulation of RFO synthesis when required [19].

The genus *Citrus* includes several economically important species, such as *C*. *sinensis* (sweet oranges), *C*. *reticulata* (mandarins and tangerines), *C*. *limon* (lemons/limes), *C*. *grandis* (pummelos) and *C*. *paradisi* (grapefruits). *Citrus* plants are cultivated mainly in semiarid regions of tropical and subtropical countries, where they constantly face severe environmental stresses such as drought and high salinity, and occasionally low temperatures and freezes, resulting in significant damage and economic losses. Accumulation of raffinose concomitant with the induction of a raffinose synthase gene homologous to the *Arabidopsis SIP1* was previously reported in roots of drought-stressed mandarin [20]. Based on these results, the authors postulated that raffinose is an important osmolyte accumulating during drought stress in citrus. In another study, transcripts encoding enzymes related to the RFO metabolism, including a GolS isoform, were identified in citrus plants infected with ‘*Candidatus* Liberibacter americanus’ (CaLam), agent of the most destructive citrus disease worldwide, the Huanglongbing (HLB) [21]. The authors postulated that RFOs could act as potent antioxidants to minimize the oxidative stress occurring near to the necrotic sieve elements formed during *Ca*Lam infection. More recently, the improved tolerance of Cleopatra mandarin (*Citrus reshni*) to drought and heat stress combination provided by the Carrizo citrange (*Poncirus trifoliata* × *Citrus sinensis*) rootstock was associated to its ability to induce the accumulation of protective compounds, such as raffinose and galactinol, in the scion [22].

Given these previous findings, the present investigation was designed to identify *GolS* genes in citrus through a genome-wide analysis of the reference genome sequences available for *Citrus sinensis* and *C*. *clementina* [23, 24] and characterize their potential role in stress tolerance. Their sequences, evolutionary relationships, putative functions and expression patterns in various tissues and in response to abiotic stresses and ‘*Ca*. Liberibacter asiaticus*’* infection were examined. Transgenic tobacco plants overexpressing *CsGolS6* were generated and analyzed. Our results provide evidence that GolS has a conserved role in citrus plants and CsGolS6 plays a function in stress tolerance.

## Materials and methods

### Identification and sequences analysis

The InterPro Entry IPR030515 (Galactinol synthase) was used as a keyword to search the *Citrus sinensis* genome sequence in the Phytozome database (https://phytozome.jgi.doe.gov/pz/portal.html#!info?alias=Org_Csinensis). The *Arabidopsis thaliana* GolS protein sequences [12] were retrieved from TAIR (http://www.arabidopsis.org/) and used to align the *C*. *sinensis* genome sequence assembly available in Phytozome by using the TBLASTN tool. After merging the results, unique entries (with unique locus ID) were identified to remove the redundancy. The resulting sequences were manually inspected for the presence of characteristic and functionally important GolS domains and motifs. We have carried out the identification of *C*. *clementina GolS* genes by searching its genome data available in the Phytozome database (http://phytozome.jgi.doe.gov/pz/portal.html#!info?alias=Org_Cclementina), as outlined for *C*. *sinensis*. Multiple sequence alignments of the deduced amino acid sequences of *C*. *sinensis* and *A*. *thaliana* were carried out using the ClustalW [25] default alignment parameters. The dendrogram was generated by the MEGA 6 program [26] using the Neighbor-Joining (NJ) method [27] and bootstrap analysis (1,000 replications).

Information about coding sequence (CDS), full-length sequence and predicted amino acid sequence was obtained for each *C*. *sinensis GolS* gene from the Phytozome database. The PROTPARAM tool available on the Expert Protein Analysis System (ExPASy) proteomics server (www.expasy.ch/tools/protparam.html) was used to predict GRAVY (grand average of hydropathy) and molecular weight of the deduced amino acid sequences. Subcellular localization of the *C*. *sinensis* GolS proteins were predicted using the WoLF PSORT tool (http://www.genscript.com/psort/wolf_psort.html). Conserved motif analysis was performed by using the MEME tool (https://meme-suite.org/meme/tools/meme) with following parameters: maximum number of motifs to find, 5; minimum width of motif, 6; and maximum width of motif, 50. Domain analysis of CsGolS sequences was performed by pfam server (http://pfam.xfam.org).

One kb upstream region from the translation start site was obtained from all the *C*. *sinensis GolS* genes and subsequently analysed in the PlantCARE server (https://bioinformatics.psb.ugent.be/webtools/plantcare/html/) to identify the presence of *cis*-acting regulatory elements in their promoter regions. The physical locations of *C*. *sinensis GolS* were determined by confirming the starting position of all genes on each chromosome, using BLASTN searching against the local database of the *Citrus sinensis* Annotation Project (CAP; http://citrus.hzau.edu.cn/orange/) [28]. The gene loci were plotted on the sweet orange chromosomes using the MapChart software [29]. The exon/intron gene structures were constructed using the Exon-Intron Graphic Maker (http://wormweb.org/exonintron).

Expression patterns of *C*. *sinensis GolS* in different tissues, i.e. callus, leaf, flower and fruit (flesh tissue), were obtained from RNA-Seq data available at the CAP database [28].

### Plant materials and stress treatments

Two-year-old sweet orange [*Citrus sinensis* (L.) Osbeck var. Westin] plants grafted on Rangpur lime (*Citrus limonia* Osbeck), a rootstock highly resistant to drought, were used in the drought stress experiment. The drought stress experiment was carried out under greenhouse conditions, with the plants grown in plastic pots of 45 L containing a mixture of Oxisol and washed sand (3:1 ratio) and subjected to control (leaf predawn water potential between -0.2 to -0.4 MPa) or drought stress (leaf predawn water potential of -1.5 MPa) treatments, as previously described [30].

For the in vitro stress tolerance assays, sweet orange [*C*. *sinensis* (L.) Osbeck var. Valencia] seeds were *in vitro* germinated as described by de Oliveira et al. [31]. Twenty-days-old nucellar and uniform seedlings were selected and transferred to MS medium containing 150 mM NaCl (Merck, Darmstadt, Germany) or only MS medium (control). Each treatment consisted of fifteen biological (plant) replicates. Leaves and roots were harvested 20 days after the treatments and immediately frozen in liquid nitrogen and stored at -80°C.

Plant infection with ‘*Candidatus* Liberibacter asiaticus’ was performed as reported in Fan et al. [32] and Martins et al. [33]. Briefly, two-year-old seedlings of rough lemon (*C*. *jambhiri Lush*.) and sweet orange (*C*. *sinensis L*. *Osbeck*) were graft-inoculated with bud wood from ‘*Ca*. L. *asiaticus*’ infected ‘Carrizo’ citrange (*C*. *sinensis L*. *Osbeck × P*. *trifoliata L*. *Raf*.) trees kept under greenhouse conditions. Plants were grafted with bud wood from healthy Carrizo trees. Plants were maintained in a Center for Disease Control-approved and secured greenhouse at the University of Florida, Citrus Research and Education Center, Lake Alfred. Three biological replicates were used for each citrus species and treatment. Quantitative real-time PCR (qPCR) was performed to confirm the presence of ‘*Ca*. L. *asiaticus’* in the inoculum source and inoculated plants as described in Li et al. [34]. Four fully expanded leaves were collected individually from ‘*Ca*. L. *asiaticus’* inoculated and mock-inoculated plants (control) of each citrus species at 0, 7, 17, and 34 weeks after inoculation (WAI), and immediately frozen in liquid nitrogen. Three biological replicates were produced for each condition. In total, 12 plants with 48 leaf samples were collected (2 *Citrus* species x 2 treatments x 3 replicates x 4 time points of infection).

Stress tolerance of wild-type (WT) and *CsGolS6*-overexpressing transgenic tobacco (*Nicotiana tabacum* cv. Havana) was examined under in vitro and greenhouse conditions. WT and transgenic (T_0_ generation) plants were grown in plastic pots of 15 L containing a mixture of soil and washed sand (ratio 3:1), in a greenhouse for three months. Seeds were then collected and germinated on MS (Murashige and Skoog) medium for the WT and MS medium + 50 mg L^-1^ kanamycin (Sigma, St. Louis, USA) for the transgenic lines. Ten-day-old WT and transgenic (T_1_ generation) seedlings were removed from germination medium and transferred to MS medium only (control treatment) or MS medium containing 20% PEG 6000 (Carbowax 6000, Union Carbide Corporation) or 150 mM NaCl (Merck, Darmstadt, Germany). Each treatment contained three replicates composed of fifteen seedlings for each WT and transgenic lines. The fresh weight (FW) of individual seedlings was determined 20 days after the treatments.

Uniform plants of WT and transgenic (T_2_ generation) lines containing an average of 10 to 15 leaves were used in the drought stress experiment. Plants were acclimatized to greenhouse conditions (25±4°C, 16 h of light and RH oscillating between 80 and 95%) for 45 days. They were grown in plastic pots of 20 L, containing a mixture of soil and washed sand (ratio 3:1). Thereafter, the pots were closed with aluminum foil to prevent water loss by evaporation and a set of 300 plants (5 plant lines x 4 treatments x 15 biological replicates) were subjected to the following treatments: (i) irrigated control, in which plants were maintained at leaf predawn water potential values of -0.2 to -0.4 MPa by daily irrigation, (ii) drought, in which the plants were exposed to a progressive soil water deficit until their leaves reach predawn water potential values of -1.6 to -2.0 MPa and (iii) rehydration, in which plants recovered their leaf predawn water potential values to-0.2 to -0.4 MPa by irrigation after the drought stress treatment. Leaf predawn water potential was measured with a Scholander-type pressure pump (m670, Pms Instrument Co., Albany, USA), between 2 AM and 4 AM, using the third fully expanded and mature leaf from the apex of each plant. Leaf relative water content (RWC) and gas exchange analyses, including the net CO_2_ assimilation rate (*A*), stomatal conductance to water vapor (*gs*) and leaf transpiration rate (*E*), which were performed in the morning (08:00–09:00 AM) using a portable photosynthesis system LI-6400 (Li-Cor) equipped with an artificial light resource (6400-02B RedBlue), were carried out as previously described [35].

### RNA extraction and expression analysis of *CsGolS*

Total RNA extraction, cDNA synthesis and qPCR analysis were performed as previously described [33]. Primer sequences are shown in Table A in S1 File. Glyceraldehyde-3-phosphate dehydrogenase C2 (*GAPC2*) was used as an internal reference gene to normalize expression among the different samples [36]. A pool of three biological replicates that were individually validated was used for gene expression analysis. The heatmap of expression of plants infected with ‘*Ca*. Liberibacter *asiaticus*’ was generated using R 3.1.0 software.

### *CsGolS6* cloning and generation of transgenic tobacco

The coding sequence of *CsGolS6* was amplified from leaves of drought-stressed ‘Rangpur’ lime by RT-PCR, using the primers 5’-ATGGCCCCTGATATCACCCCC-3’ and 5’-CTAAGCAGCAGACGGGGCGG-3’, and cloned in sense orientation into the *Sal*I/*Not*I sites of pUC118 under the control of the *CaMV 35S* promoter (*35S-P*) and terminator (*35S-T*) sequences. The resulting *35S-P*::*CsGolS6*::*35S-T* expression cassette was then removed from the pUC118 vector by digestion with *Pst*I and inserted into the same restriction site of pCAMBIA 2301. pCAMBIA 2301 vector contains the neomycin phosphotransferase II (*nptII*) selectable marker gene and the beta-glucuronidase (GUS) *uid*A scorable marker gene driven by the *35S-P*. This vector was introduced into the EHA105 *Agrobacterium tumefaciens* strain by direct DNA uptake, and used for *Agrobacterium*-mediated genetic transformation of tobacco as described previously [35].

Several independent transgenic lines (T_0_ generation) derived from distinct transformation events were transferred to soil and grown under standardized greenhouse conditions to generate seeds by self-pollination. T_1_ seeds were collected individually from each T_0_ plant and screened on plates containing MS medium + 50 mg L^-1^ kanamycin for analysis of antibiotic resistant:susceptible segregation ratio. PCR screening was used to distinguish between transgenic and WT plants as previously described [35]. qPCR analysis to assess the *CsGolS6* expression levels in the different transgenic lines was performed as described above.

### Leaf anatomy

Leaf anatomy of WT and *CsGolS6-*overexpressing transgenic (T_2_ generation) lines was analyzed as previously described [35]. Briefly, leaf cross-sections were collected from the third leaf from the apex of plants maintained for 45 days under greenhouse conditions. Samples of the median portion of the leaf blade were fixed in FAA 70 [37] and stored in 70% (v/v) etanol. Samples were then dehydrated in a graded ethanol series and embedded in methacrylate (HistoResin, Leica), as described by Feder and O’Brien [38]. Material was sectioned at 10 μm using a rotary microtome and sections were stained with toluidine blue [38]. Anatomical features were measured, including the adaxial epidermis, abaxial epidermis, palisade parenchyma and spongy parenchyma. Observations and photographic documentation were done using a photonic microscope Leica DM 300 (Leica Microsystems, Wetzlar, Germany). Three biological replicates were used, each containing three slides.

### Primary metabolite profiling

Extraction of primary metabolites, derivation standard addition and sample injection for GC-MS analyzes were performed according to Lisec et al. [39], with modifications. Extraction of primary metabolites was performed in 10 mg of lyophilized leaf tissue with the addition of 60 μl per sample of the extracting solution containing methanol:chloroform:water (2.5:1:1 v/v/v) and ribitol internal standard (0.2 mg ml^-1^ in H_2_O). A volume of 1.5 ml of the extraction mix was added to the plant material and homogenized by vortexing for 10 s, incubated at 4°C for 30 min and then centrifuged at 14,000 rpm for 5 min. A volume of 0.75 ml of ultrapure H_2_O was added to 1 ml of supernatant. The mixture was then homogenized by vortexing (15 s) and centrifuged at 14,000 rpm for 15 min. The polar top phase containing the primary polar metabolites were collected and dried in SpeedVac without heating. Forty μl of methoxyamine hydrochloride (20 mg ml^-1^ in pyridine) was added to the samples, which were then incubated for 2 h at 37°C, under agitation of 950 rpm. After that, 70 μl of a solution containing 1 ml of N-methyl-N-trimethylsilyltrifluoroacetamide (MSTFA) and 20 μL of FAME was added to the samples, which were again incubated for 30 min at 37°C under agitation of 950 rpm. The volume of 100 μl was transferred to vials to be applied to GC-MS.

The GC-MS TruTOF system was composed by a autosampler Split/Splitless, Agilent 7890A gas chromatograph and a mass spectrometer LECO TruTof. Chroma TOF software 4.4 (Leco) and TagFinder 4.0 [40], supplied with the library containing the retention time and fragmentation pattern of the primary metabolites of Golm metabolome database, were used to evaluate chromatograms and mass spectra. Once identified, the relative values of metabolites were normalized by the ribitol internal standard, sample weight and the average of the relative value of irrigated (control) WT. Heatmap was generated using the Heatmapper Tool (http://www.heatmapper.ca/expression/) with the parameters scale type ‘column’, clustering method ‘complete linkage’ and distance measurement method ‘Manhattan’.

### Statistical analysis

Statistical analysis was carried out with the software BIOESTAT (Universidade Federal do Pará, Brazil), which tested the experiments as a completely randomized design. Statistical differences were assessed based on the analysis of variance (ANOVA) and means were separated by Dunnett or Student’s *t* test, with a critical value of *P* ≤ 0.05.

## Results

### Identification and sequence analysis of citrus *GolS* genes

Eight *GolS* related genes were identified from the *C*. *sinensis* and *C*. *clementina* genome sequences available in the Phytozome database. The retrieved sequences were inspected and confirmed to have the characteristic conserved GT8 (PF01501) domain (Fig A in S1 File). *C*. *sinensis GolS* (*CsGolS*) genes encode proteins ranging from 203 (23.7 kDa) to 637 (73.6 kDa) amino acids in length and pI values ranging from 4.78 to 9.54, with most of them exhibiting a pI of <7 (Table 1). All the predicted proteins have a negative grand average hydropathy (GRAVY) score, suggesting that they are hydrophilic proteins, and are predicted to be localized in the cytoplasm (Table 1). Motif analysis found five conserved motif sequences, with motif 1–3 related with GT8 (PF01501) domain structure, motif 5 with Inhibitor_I67 (PF11405) domain, and motif 4 not related to any domain structure (Fig B in S1 File).

**Table 1:** Characteristics of genes encoding citrus GolS proteins.

Gene	*C*. *sinensis* ID (Phytozome)	*C*. *sinensis* ID (CAP)	*C*. *clementina* homolog ID (Phytozome)	Chromosome location	CDS length (number of exons)	Polypeptide length (MW)	pI	GRAVY	Predicted subcellular localization
*CsGolS1*	**orange1.1g006648m**	Cs1g05500	Ciclev10025272m	chr1:5,229,069..5,232,119	1,914 (4)	637 (73.62kDa)	6.61	-0.353	Cytoplasm
*CsGolS2*	**orange1.1g043696m**	Cs7g19860	Ciclev10003328m	chr7:15,999,350..16,002,178	1,566 (2)	521 (60.54kDa)	5.95	-0.358	Cytoplasm
*CsGolS3*	**orange1.1g007705m**	Cs3g23600	Ciclev10000658m	chr3:25,840,038..25,843,105	1,779 (2)	592 (69.36kDa)	9.04	-0.348	Cytoplasm
*CsGolS4*	**orange1.1g037463m**	Cs3g13670	Ciclev10000664m	chr3:18,022,540..18,025,263	1,035 (3)	344 (40.74kDa)	6.54	-0.497	Cytoplasm
*CsGolS5*	**orange1.1g041855m**	Cs3g13720	Ciclev10004021m	chr3:18,115,719..18,118,535	609 (2)	203 (23.68kDa)	9.54	-0.393	Cytoplasm
*CsGolS6*	**orange1.1g024367m**	Cs3g15460	Ciclev10001308m	chr3:19,579,954..19,581,767	807 (2)	268 (30.85kDa)	5.71	-0.103	Cytoplasm
*CsGolS7*	**orange1.1g020230m**	Cs9g01710	Ciclev10005406m	chr9:468,115..470,386	990 (4)	329 (37.94kDa)	4.78	-0.188	Cytoplasm
*CsGolS8*	**orange1.1g019647m**	orange1.1t01729	Ciclev10021027m	chrUn:27,794,711..27,796,299	1,014 (3)	337 (38.27kDa)	5.18	-0.193	Cytoplasm

**Fig 1:**
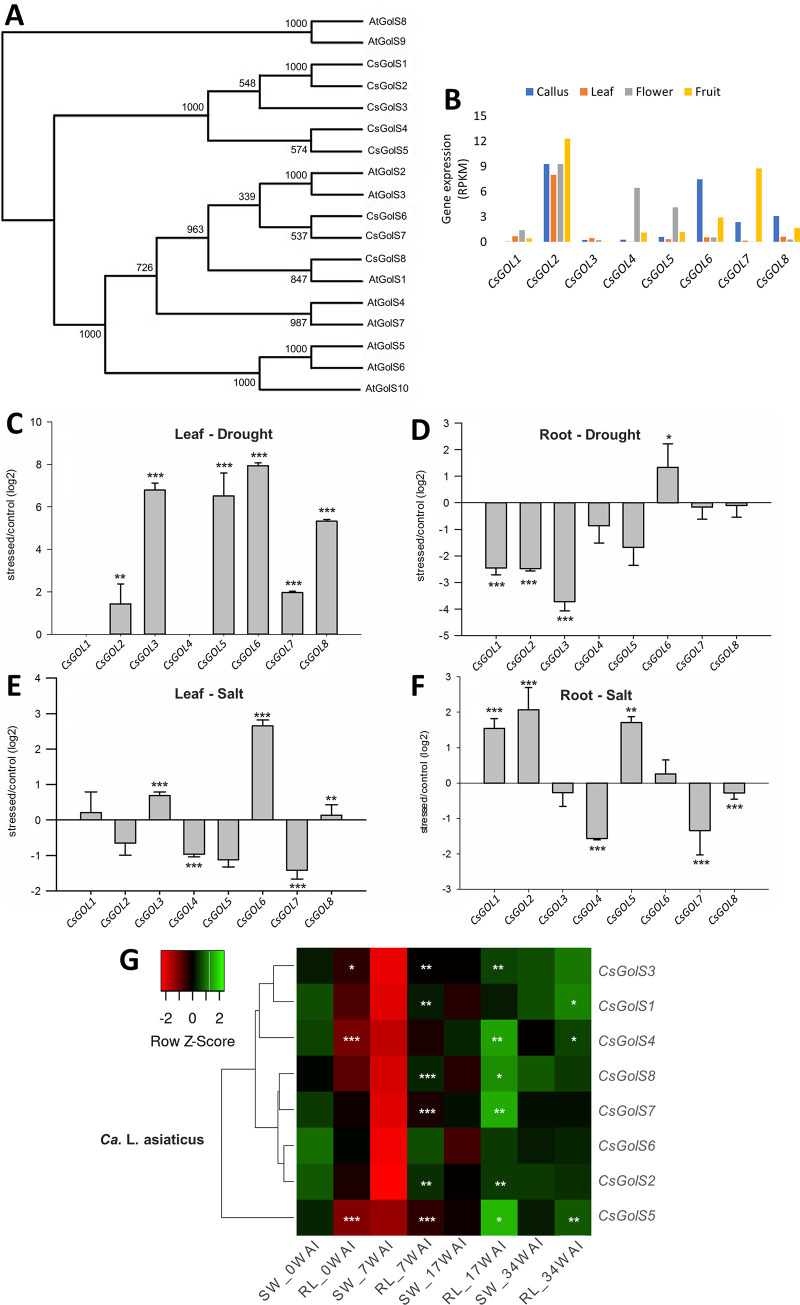
Phylogeny and expression of *C*. *sinensis GolS* (*CsGolS*) genes. (A) Phylogenetic relationships of CsGolS amino acid sequences with those of *Arabidopsis thaliana* (At). (B) RNA-Seq data of *CsGolS* expression in the different *C*. *sinensis* tissues. RPKM: reads per kilobase per million mapped. (C-G) Expression analysis of *CsGolS* genes in response to drought (C, D) and salt (E, F) stresses and ‘*Ca*. L. asiaticus’ infection (G). Ratios (log2) of relative mRNA levels in leaves and roots between stressed and control plants of sweet orange (C-F) and in leaves between infected and control plants of rough lemon (RL) and sweet orange (SW) at 0, 7, 17, and 34 WAI (G), as measured by qPCR. *GAPC2* was used as an endogenous control. Data are means ± SE from three biological replicates. *,**,***Significantly different from control treatment (C-F) or sweet orange at the respective WAI (G) by the Student’s *t* test at *P* ≤ 0.05, *P* ≤ 0.01 or *P* ≤ 0.001, respectively.

Phylogenetic analysis revealed that CsGolS amino acid sequences are highly homologous with each other as well as with the AtGolS (Fig 1A). CsGolS1-5 have no close *AtGolS* homologs. CsGolS6-7 are orthologs of AtGolS2-3, while CsGolS8 is an ortholog of AtGolS1. BLAST searches against the genome sequence of *C*. *clementina* showed that there is a *GolS* ortholog in its genome for each *C*. *sinensis GolS* gene (Table 1).

The exon-intron structure of the *CsGolS* genes was analyzed using the *C*. *sinensis* gene models annotated in the Phytozome database. *CsGolS2*, *-3*, -*5* and -*6* had a single intron and two exons (Fig C in S1 File). On the other hand, *CsGolS4* and -*8* had two introns and three exons. *CsGolS1* and -*7* contained three introns and four exons.

Analysis of the 1 kb upstream promotor region of *CsGolS* genes revealed the presence of several *cis*-acting regulatory elements, which were classified into five categories according to their biological function: (1) stress response, (2) hormone response, (3) wounding response, (4) light response, and (5) plant development-related elements (Fig D in S1 File). Among these *cis*-acting regulatory elements, stress-responsive elements were the most abundant and included CAAT-box, MYB, MYC, STRE, LTRE, ARE, and DRE/CRT. ABRE was the most abundant *cis*-acting regulatory element related to hormonal regulation, while G-box was the predominant one related to light responsiveness (Fig D in S1 File). These results suggest that *CsGolS* are involved in various abiotic and biotic stress- and light-induced responses.

The positions of *CsGolS* were mapped on the *C*. *sinensis* chromosomes by homology searches against the *C*. *sinensis* genome assembly available in the CAP database (Fig E in S1 File). *CsGolS* loci were distributed throughout four *C*. *sinensis* chromosomes (chr 1, 3, 7 and 9), with chromosome 3 carrying four of them. The closely related *CsGolS* isoforms *CsGolS4* and -*5* were mapped on close positions (~90 kb proximity to each other) in the chromosome 3, indicating that they are tandem duplicates. *CsGolS8* was not exactly located on any chromosome (ChrUN) because of an incomplete physical map for *C*. *sinensis*.

### Expression of *CsGolS* genes in different tissues and in response to abiotic and biotic stress conditions

Expression analysis in different tissues from RNA-seq data indicated that only *CsGolS2* was relatively abundantly expressed across all tissues analyzed (Fig 1B). On the other hand, a tissue-specific expression was observed for *CsGolS4* and -*5* in flowers, *CsGolS6* and -*8* in callus and *CsGolS7* in fruits. *CsGolS1* and -*3* transcripts were rarely detected in the different tissues tested (Fig 1B).

Expression analysis of *CsGolS* genes in response to drought and salt stresses and ’*Ca*. L. asiaticus’ infection revealed that they are differentially regulated by the different stresses. qPCR analysis showed that all the *CsGolS* genes were differentially expressed in at least one stress condition tested (Fig 1C–1G). *CsGolS2*, *-3*, *-5*, *-6*, *-7* and *-8 *were upregulated by drought stress in leaves, whereas *CsGolS6* was upregulated and *CsGolS1*, *-2*, and *-3* were downregulated by this treatment in roots (Fig 1C and 1D). *CsGolS3*, *-6* and *-8* were induced and *CsGolS4* and -*7* were repressed by salt stress in leaves, whereas *CsGolS1*, *-2* and *-5* were induced and *CsGolS4*, *-7* and -8 were repressed by this treatment in roots (Fig 1E and 1F). The time-course transcriptional analysis in response to ‘*Ca*. L. asiaticus’ infection showed that the *CsGolS* genes were downregulated at the early stage (7 WAI) of disease in the highly susceptible sweet orange, but not in the tolerant rough lemon, whose expression levels of most *CsGolS* were significantly different from those of sweet orange at this stage and also at 17 WAI (Fig 1G). *CsGolS5* was the only *CsGolS* gene significantly differentially expressed between the tolerant and susceptible citrus genotypes at all stages of infection.

### *CsGolS6*-overexpressing transgenic tobacco shows enhanced tolerance to drought and salt stresses

Our comprehensive analysis of the *CsGolS* gene family allowed us to select *CsGolS6* gene as the first *CsGolS* family member to be functionally characterized in citrus. The criteria used to choose this gene over the others were based on its (i) orthology with the stress-inducible *Arabidopsis AtGolS2-3* (Fig 1A), (ii) highest number of drought stress-responsive *cis*-acting regulatory elements ABRE and DRE/CRT present in the promoter region (Fig D in S1 File), and (iii) upregulation by drought and salt stress in leaves (Fig 1C and 1E) and by drought stress in roots (Fig 1D). Several putatively *CsGolS6*-overexpressing tobacco regenerants on kanamycin-containing medium were generated and screened for the presence of a 800-bp fragment of the *nptII* gene by PCR analysis (Fig F in S1 File). *CsGolS6* overexpression was confirmed in the PCR-positive transgenic lines by qPCR expression analysis (Fig F in S1 File). The transgenic lines expressed *CsGolS6* at relatively high and varying levels. They exhibited a normal phenotype and were fertile.

**Fig 2:**
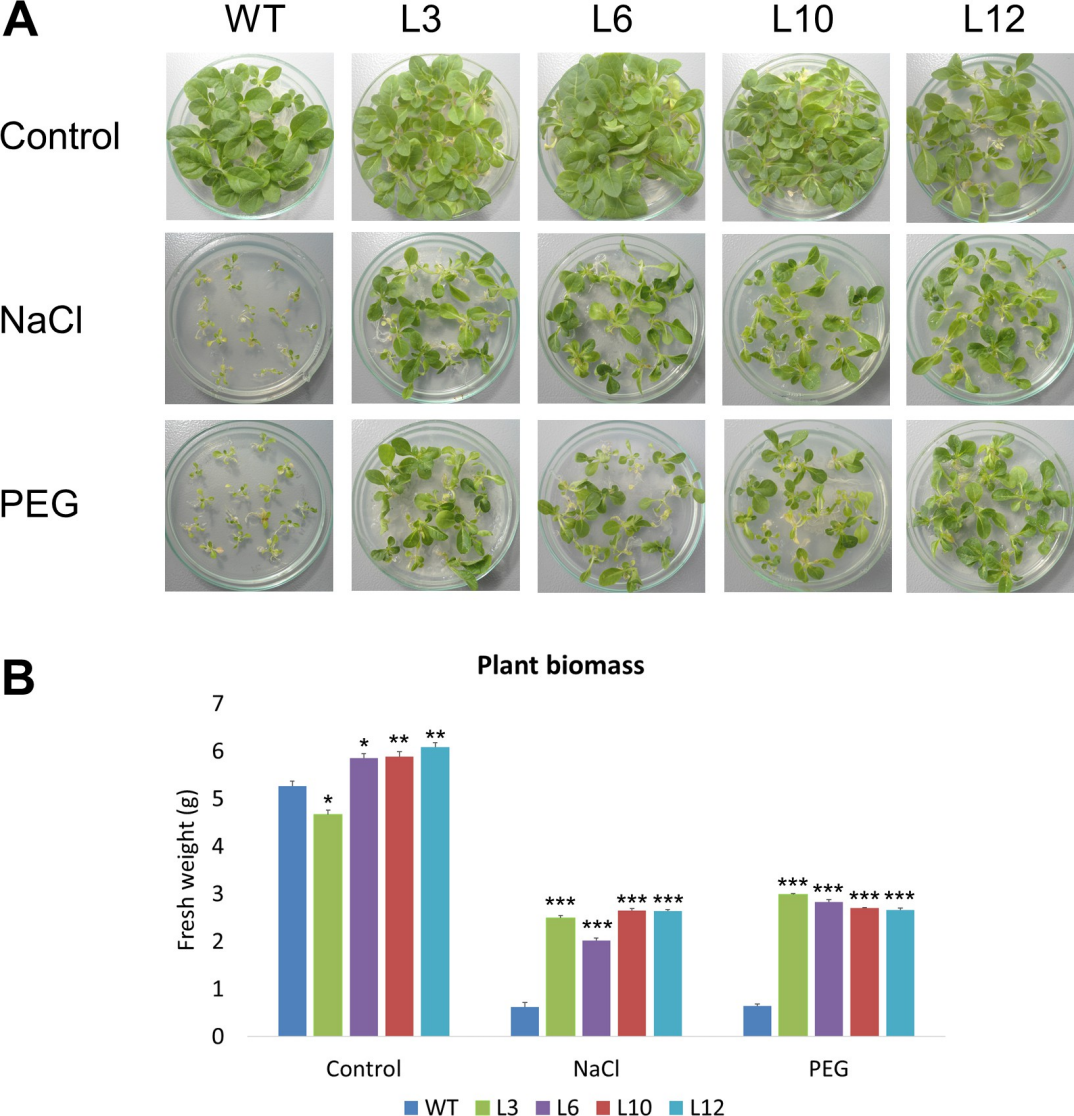
In vitro stress tolerance assay of *CsGolS6*-overexpressing transgenic tobacco plants. (A) Representative phenotypes of WT and *CsGolS6-*overexpressing transgenic (L3-12) lines grown under control, salt and PEG treatments for 20 days. (B) Seedling biomass of WT and transgenic lines under control, salt and PEG treatments for 20 days. The data are means ± SE of three technical replicates composed of fifteen seedlings for each line. *,**,***Significantly different from WT at the respective treatment by the Student’s *t* test at *P* ≤ 0.05, *P* ≤ 0.01 or *P* ≤ 0.001, respectively.

In order to evaluate the effects of the *CsGolS6* overexpression on stress tolerance of the transgenic plants, WT and transgenic lines were subjected to in vitro stress tolerance assays using PEG and NaCl treatments. The representative phenotypes of WT and transgenic plants under control and stress treatments are shown in Fig 2A. The results revealed no significant differences in biomass between WT and transgenic lines in the control treatment (Fig 2B). However, seedlings from the transgenic lines have more than 3-× higher biomass than the WT under PEG and salt treatments. Besides the severely retarded growth, the stressed WT plants showed severe symptoms of wilting and chlorosis (Fig 2A).

### *CsGolS6-*overexpressing transgenic tobacco shows improved physiological and growth performance under drought stress conditions

**Fig 3:**
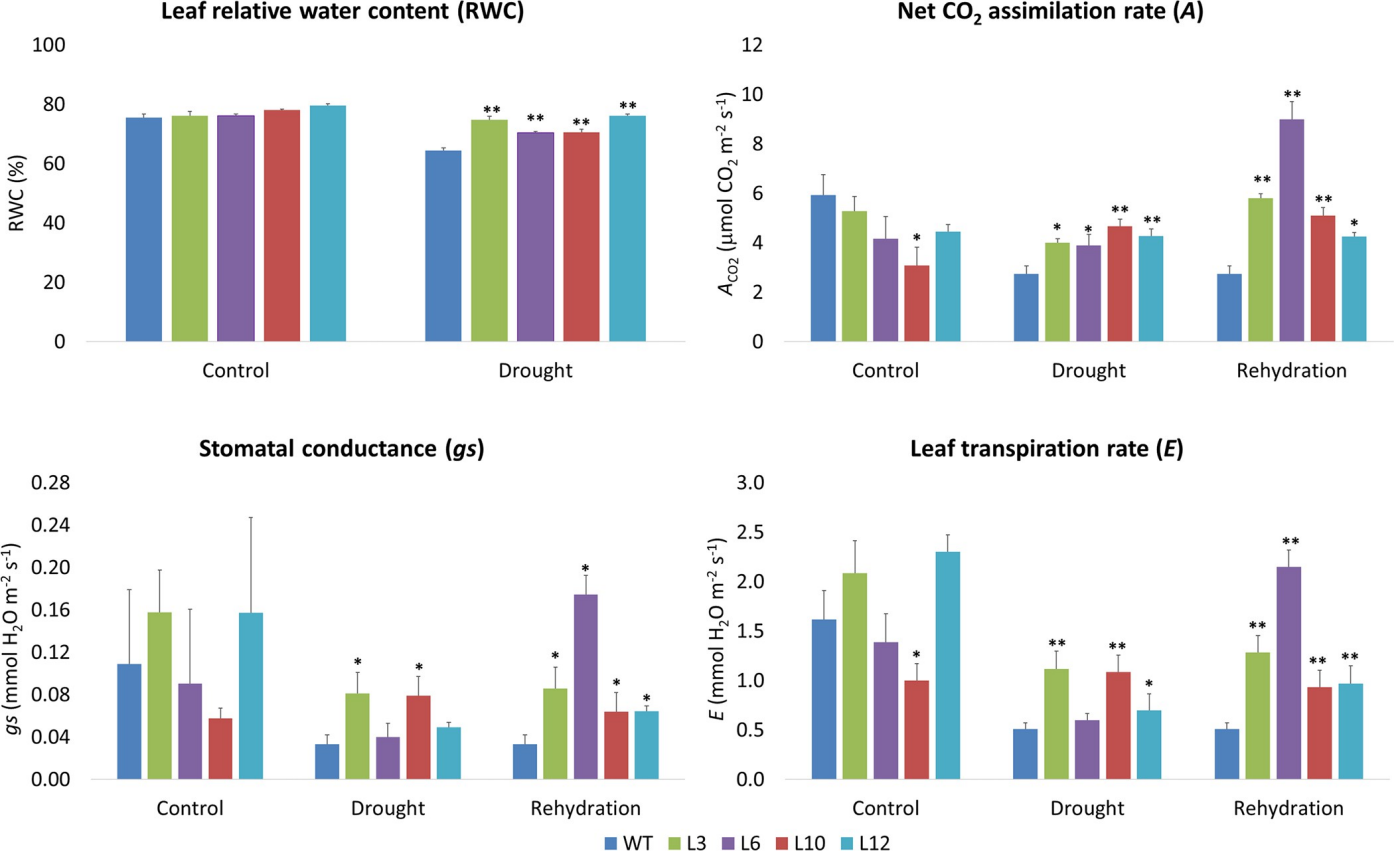
Physiological analysis of WT and *CsGolS6-*overexpressing transgenic tobacco plants under different water regimes in greenhouse conditions. WT and transgenic (L3-L12) plants were exposed to three water regimes: (i) control (leaf water potential at -0.3 a -0.5 MPa), (ii) drought (leaf water potential at -1.5 a -2.0 MPa) and (iii) rehydration (leaf water potential at -0.3 a -0.5 MPa after a cycle of drought stress). The data are means ± SE of ten biological replicates. *,**Significantly different from WT at the respective water treatment by the Dunnett test at *P* ≤ 0.05 or *P* ≤ 0.01, respectively.

The *CsGolS6*-overexpressing transgenic and WT plants were grown in greenhouse conditions and then exposed to drought stress by decreasing the water supply gradually for 45 days. Physiological variables such as leaf RWC, *A*, *gs* and *E* were measured during the dehydration and rehydration cycle. No significant differences in RWC, *A*, *gs*, *E* were observed between transgenic and WT plants in control (irrigated) condition (Fig 3). On the other hand, the *CsGolS6*-overexpressing transgenic plants were able to maintain significantly higher values of RWC, *A*, *gs* and *E* than the WT under drought and after rehydration (Fig 3). Under such conditions, the transgenic plants exhibited as much as 10–18% increased RWC values and 18–570% increased *A*, *gs* and *E* values in comparison with WT.

**Fig 4:**
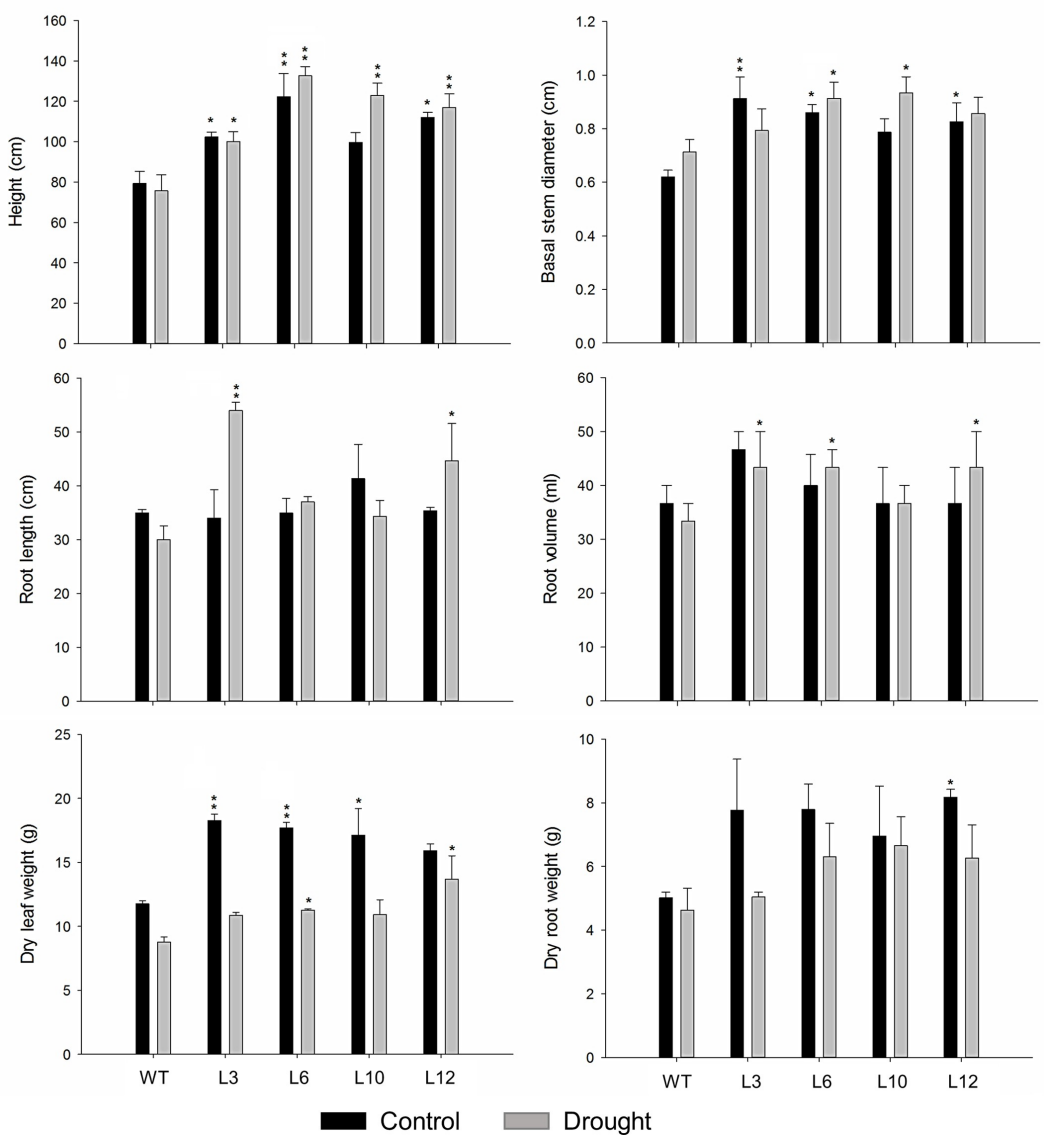
Growth performance of WT and *CsGolS6-*overexpressing transgenic tobacco under control (irrigated) and drought-stress conditions. WT and transgenic (L3-L12) plants grown under greenhouse conditions were exposed to a progressive soil water deficit (drought) until reach leaf water potentials of ~-2.0 MPa, or maintained at leaf water potentials of -0.3 to -0.5 MPa as the control treatment. The data are means ± SE of ten biological replicates. *,**Significantly different from WT at the respective water treatment by the Dunnett test at *P* ≤ 0.05 or *P* ≤ 0.01, respectively.

Analysis of the growth performance of the *CsGolS6*-overexpressing transgenic and WT plants was carried out in both control and drought stress conditions. Except for L10 in the control treatment, all the transgenic plants were significantly taller than the WT in both control and drought conditions (Fig 4; Fig G in S1 File). For other growth parameters evaluated, such as basal stem diameter and leaf dry weight, the transgenic plants showed a tendency to exhibit increased values as compared with the WT in both water treatments, although the differences were non-significant in some cases (Fig 4). Considering the root variables, no significant differences were found between WT and transgenic plants under control condition. However, the transgenic plants tended to exhibit an increased root length and volume under drought (Fig 4; Fig G in S1 File).

**Fig 5:**
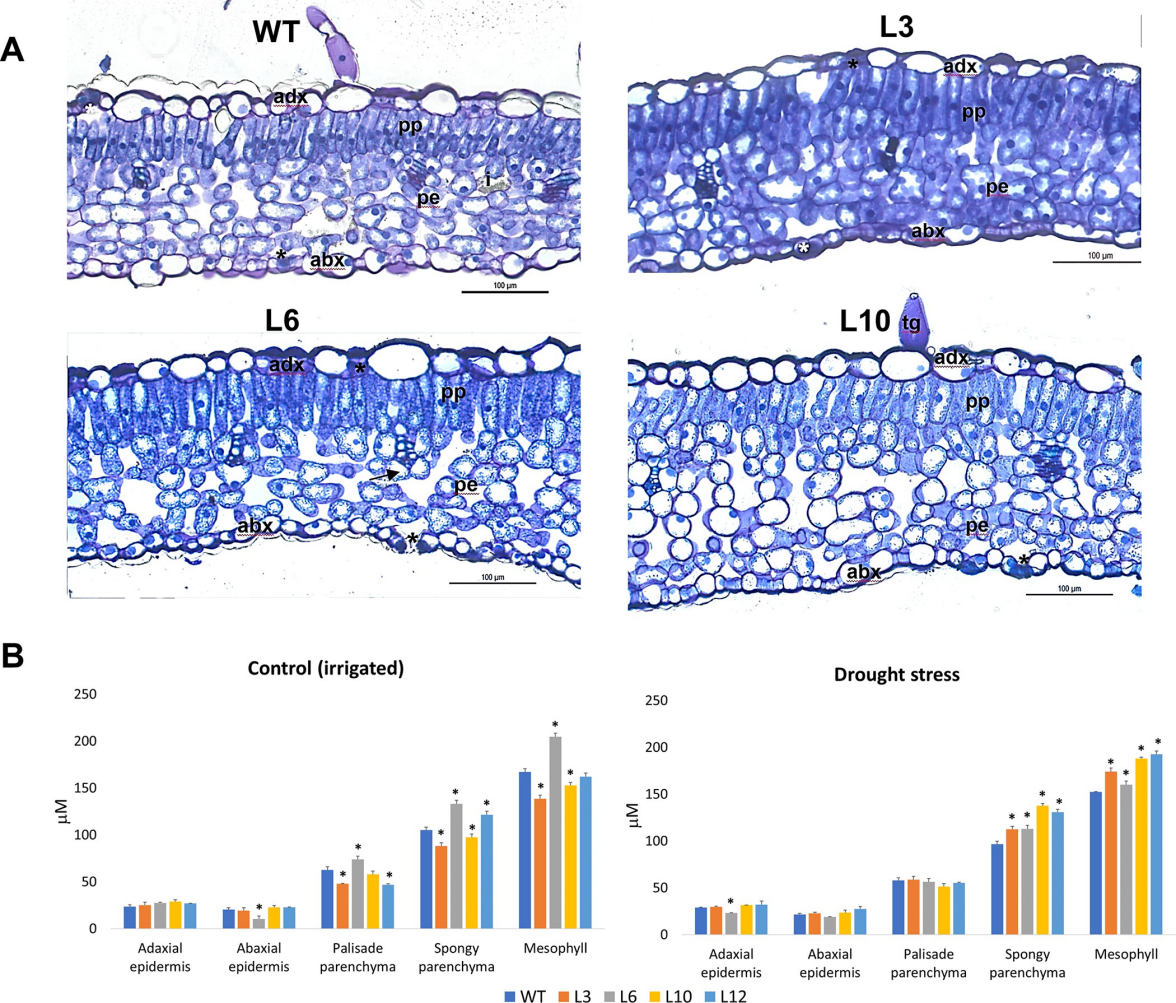
Anatomical analysis of leaf cross-sections of WT and *CsGolS6-*overexpressing transgenic plants under control (irrigated) and drought stress conditions. (A) Leaf cross-section of WT and *CsGolS6-*overexpressing transgenic (L3, L6 and L10) plants subjected to drought stress, as observed under photonic microscope. The mesophyll region is shown. Magnification bars represent 100 μm. Adaxial side (adx), abaxial side (adx), palisade parenchyma (pp), spongy parenchyma (pe), glandular trichomes (tg), stomata (*), vascular tissue (arrow) and idioblasts (i). (B) Quantitative analysis of the leaf anatomy of WT and transgenic (L3, L6, L10 and L12) plants subjected to control (irrigated) and drought stress treatments. The data are means ± SE of three technical replicates, each containing three slides composed for each plant line. *Statistically significant differences between WT and transgenic plants by the Dunnett test at *P* ≤ 0.05.

Analysis of the anatomy of leaf cross-sections of WT and transgenic plants showed that the spongy parenchyma cells were larger in the transgenic than in the WT plants under drought condition (Fig 5A and 5B). Such an increase contributed to the increased thickness of the mesophyll in the transgenic plants under drought (Fig 5B). In control conditions, the results were more variable, with two transgenic lines (L3 and L10) showing a reduction and one showing an increase (L6) of parenchyma cells and mesophyll (Fig 5B).

### Primary metabolite profiling of *CsGolS6*-overexpressing transgenic plants

**Fig 6:**
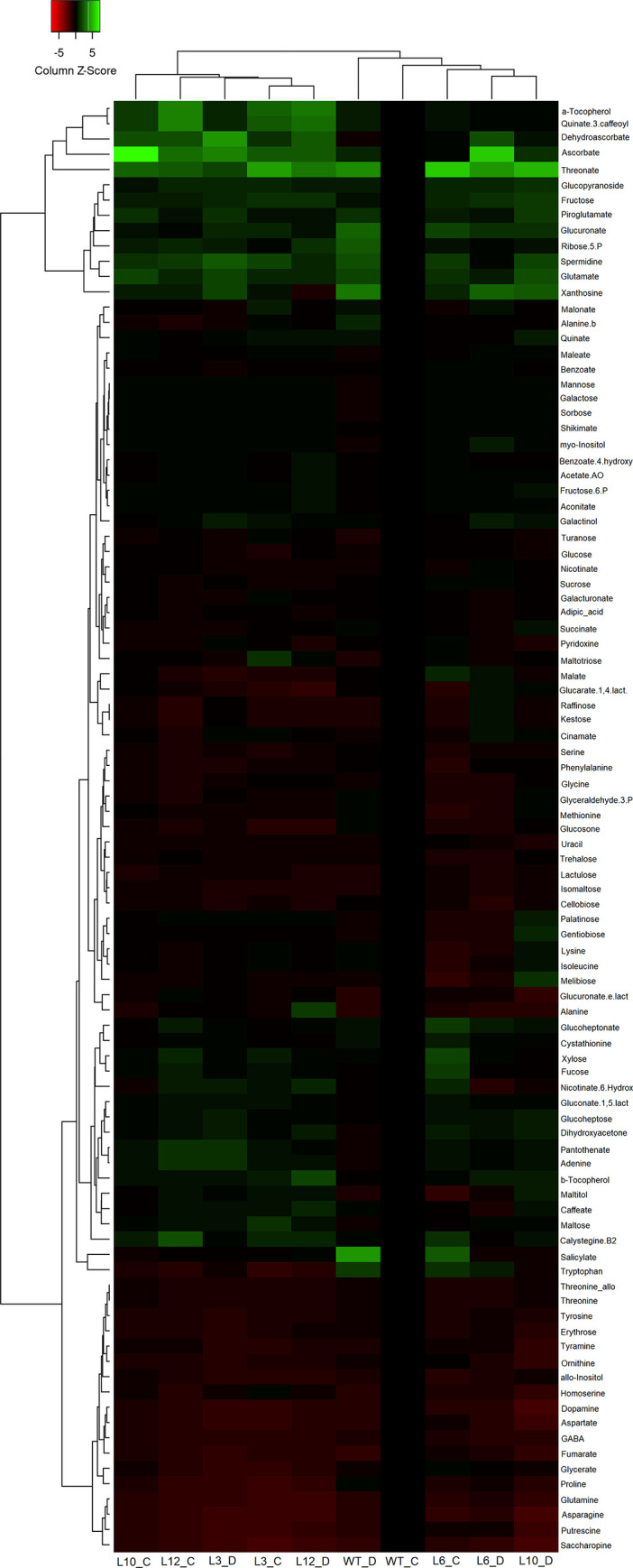
Primary metabolite profiles of WT and *CsGolS6-*overexpressing transgenic tobacco under control (C) and drought (D) conditions. Green and red colors represent, respectively, increase and decrease of metabolites using a false-color scale. Values are the means of three biological replicates.

Metabolite profiling of leaves of WT and *CsGolS6*-overexpressing transgenic plants accurately quantified 95 metabolites of known chemical structure in every chromatogram. These compounds included most plant amino acids, organic acids, TCA cycle metabolites, sugars, sugar alcohols, secondary metabolites, polyamines, among others. There were no significant variations in the levels of galactinol between WT and transgenic plants, whereas the raffinose levels were significantly lower (*P* ≤ 0.05) in two out of the four transgenic lines in comparison to the WT (Fig 6 and Table B in S1 File). On the other hand, a significant increase (*P* ≤ 0.05) in the levels of ascorbate, dehydroascorbate, threonate, alfa-tocopherol and spermidine, as well as a significant decrease in the levels of tyramine, kestose, dopamine and putrescine, were observed in at least two out of the four transgenic lines, as compared to the WT (Fig 6 and Table B in S1 File).

## Discussion

The number of *GolS* genes identified in the genomes of *C*. *sinensis* and *C*. *clementina* is similar to that found in other plant species, such as eight in *A*. *thaliana* [16] and *Malus × domestica* [41] and nine in *Populus trichocarpa* [42] and *N*. *tabacum* [43]. GolS has been characterized as a plant-specific protein subfamily among the GT8 group of enzymes derived from a single ancestral sequence, which has been lost or differently evolved in the different plant species [2]. In fact, five CsGolS sequences (CsGolS1-5) were found to have no close homologs in *Arabidopsis* (Fig 1A), suggesting that they evolved by gene duplication events after the divergence of the *Arabidopsis* and *Citrus* lineages. Consistent with this idea is the fact that four out of the eight *CsGolS* genes identified in the present study are located in the chromosome 3 (Table 1; Fig E in S1 File), with two of them (*CsGolS4* and -*5*) arranged as tandem duplicates according to defined criteria [44].

The *CsGolS* genes contain three or few introns (Fig C in S1 File) like most of the *GolS* family members [41, 43, 45]. Their predicted protein sequences also have the characteristic GT8 domain (Figs A and B in S1 File), which consists of two loosely defined regions: the N-terminal 100–120 residues representing the nucleotide diphosphate (NDP)‐sugar donor (UDP-galactose)‐binding site and the C-terminal region that binds to the acceptor molecule (L-*myo*-inositol) [2]. The CsGolS proteins were predicted to have a cytoplasmic subcellular location (Table 1), consistent with their characterization as an extravacuolar enzyme [46]. In addition, most CsGolS proteins have a pI of <7 (Table 1), a feature which may explain why GolS is enzymatically active under acidic conditions [43].

Orthology prediction is helpful for assigning functional information to genes in other plant species. CsGolS6-7 are orthologs of AtGolS2-3, while CsGolS8 is an ortholog of AtGolS1 (Fig 1A). *AtGolS1* and *AtGolS2* genes were highly expressed in mature seeds and induced by drought and high-salinity, whereas the expression of *AtGolS3* was induced by cold stress and hardly detected in mature seeds [16]. Overexpression of *AtGolS1-2* increased the galactinol and raffinose contents and resulted in effective osmoprotection [16] and ROS scavenging capacity in transgenic *Arabidopsis* [12]. *AtGolS2* overexpression also improved drought tolerance in transgenic *Brachypodium* [47] and rice [48] and increased rice grain yield in the field [48]. Thus, it is expected that *CsGolS6*, *-7* and *-8* play similar functions as reported for their respective orthologs in *A*. *thaliana*.

The *cis*-acting regulatory elements found in promoter regions of the *CsGolS* (Fig D in S1 File) suggest that most of them are stress-inducible genes as reported in other plant species [12, 16, 42, 45, 49]. Consistent with these findings is the observation that all the *CsGolS* genes were transcriptionally regulated by abiotic and/or biotic stresses (Fig 1C–1G). The observed inducible expression of *CsGolS2*, *-3*, *-5*, *-6*, *-7* and *-8* by drought stress, *CsGolS1*, *-2*, *-3*, *-5*, *-6* and *-8* by salt stress and *CsGolS5* by ‘*Ca*. L. asiaticus’ infection (Fig 1C–1G) suggest a functional role for these genes in abiotic and/or HLB stress tolerance. Such a functional role is postulated to be related to the predicted RFO functions in osmoprotection [16], signal transduction [10] and/or antioxidant defense [12].

Tissue expression pattern analysis of *CsGolS* genes provided important clues about their functional role in carbon storage and transport. *CsGolS2* showed a relatively high and constitutive expression across the different tissue types analyzed, whereas a tissue-specific preferential expression was observed for *CsGolS4* and -*5* (flowers), *CsGolS6* and -*8* (callus) and *CsGolS7* (fruits) (Fig 1B). Most of the *GolS* genes show a low-level constitutive or tissue-specific expression, and specific tissue locations of RFO synthesis and related-genes expression emerge as an important control of RFO biosynthesis [1, 2]. For instance, in photosynthesizing leaves of *Ajuga reptans* there are two RFO pools: a storage pool associated with the leaf mesophyll and a transport pool associated with the phloem-loading sites [50], where RFOs are produced and loaded in the phloem [51]. These two pools rely on different *GolS* genes [18]. Based on these findings, one can speculate a functional role for CsGolS2 in carbon storage and/or transport across all tissues analyzed, whose function may be complemented or strengthened by the activity of CsGolS4-*5* in flowers, CsGolS6-8 in callus and CsGolS7 in fruits.

To assess the function of the drought and salt stress-inducible *CsGolS6* gene in stress tolerance, *CsGolS6*-overexpressing tobacco plants were generated and examined for their tolerance to drought- and salt-stress treatments. In comparison to WT, all the transgenic plants showed a significantly enhanced tolerance to drought and salt stresses (Figs 2–4). The transgenic plants were able to maintain significantly higher values of biomass under PEG and salt-stress treatments (Fig 2), and of leaf RWC, *A*, *gs*, *E* (Fig 3) and most growth variables (Fig 4; Fig G in S1 File) under drought-stress and rehydration. In addition, the transgenic plants showed a significantly increased spongy parenchyma cell size and mesophyll under drought-stress condition (Fig 5). Collectively, these data indicate that the *CsGolS6* expression contributes to plant photosynthesis, growth and mesophyll cell expansion under stress condition. Physiological processes such as stomatal conductance, photosynthesis and leaf tissue expansion can be maintained, at least partially, when turgor is able to be retained even at low soil water potentials, thus ensuring plant growth under stress conditions [9]. Similar results to ours were also observed in salt-stressed transgenic *Arabidopsis* plants overexpressing the *AtGolS2* homolog *TsGolS2* from *Thellungiella salsuginea*, which showed improved photosynthesis rates and seedling growth that were associated to their increased contents of galactinol, raffinose and α-ketoglutaric acid, a key intermediate organic acid in TCA cycle [52]. A link between RFO metabolism and the level of α-ketoglutaric acid in transgenic *Arabidopsis* and the role of this metabolic interlinking in plant abiotic stress tolerance were not investigated.

Primary metabolite profiling was used to examine the metabolic changes caused by the *CsGolS6* overexpression in leaves of transgenic plants. The transgenic plants did not exhibit increased levels of galactinol or raffinose as expected, but rather increased levels of metabolites with antioxidant properties, such as ascorbate, dehydroascorbate, alfa-tocopherol and spermidine (Fig 6 and Table B in S1 File). These results suggest a link between *CsGolS6* expression and the metabolism of these antioxidants. The interconnection between RFO and ascorbate, alfa-tocopherol and polyamine metabolic pathways might explain these results. Raffinose can be hydrolyzed to sucrose and D-galactose, by the action of the enzyme α-galactosidase (α-GAL), and sucrose can be further hydrolyzed to fructose and glucose, by invertases, or to fructose and UDP-glucose, by sucrose synthases [53, 54]. Fructose, glucose and UDP-glucose can then readly enter other metabolic pathways, including those of ascorbate via D-mannose and D-glucuronate [55], and polyamines via TCA cycle [56]. Ascorbate oxidation leads to the formation of dehydroascorbate (oxidized ascorbate), while ascorbate catabolism leads to the formation of threonate [55, 57]. Ascorbate is also able to regenerate α-tocopherol from the α-chromanoxyl radical [57]. Alternatively, CsGolS6 might have another enzymatic activity that stimulates the synthesis of these antioxidant metabolites, since it clades on a separate branch together with CsGolS7 in the phylogenetic analysis (Fig 1A). Further investigation is required to elucidate the molecular mechanism of stress tolerance mediated by CsGolS6.

## Conclusions

The data reported here show that *GolS* constitutes a relatively small and conserved family of stress-inducible genes in citrus. They also suggest that CsGolS6 plays a role in stress tolerance by increasing the levels of antioxidant metabolites. This study provides a basis to further exploring the molecular mechanisms of CsGolS6 in plant stress tolerance.

## Supporting information

S1 File (DOCX)
