# Supplementary material for: Comprehensive analysis of the GALACTINOL SYNTHASE (GolS) gene family in citrus and the function of CsGolS6 in stress tolerance
Source: PLoS One. 2022 Sep 16;17(9):e0274791. doi: 10.1371/journal.pone.0274791 (PMC9481003; doi:10.1371/journal.pone.0274791)
Supplement: S1 File — (DOCX) [file pone.0274791.s001.docx]

**Supporting Information (Martins et al.)**

**Table A.** Primers used in the qPCR analysis

| **Name** | **Locus ID** | **Primer** | **Size product (bp)** |
| --- | --- | --- | --- |
| *CsGolS1* | orange1.1g006648m | F: 5’-CCCTGCAAGGAACTCGTGAC-3’  R: 5’-CAAGAACCAACCGGAAGCTG-3’ | 98 |
| *CsGolS2* | orange1.1g043696m | F: 5’-TCAAGAGATGTGGCCCGTTT-3’  R: 5’-CCAAAAGCACATGCACTGGA-3’ | 91 |
| *CsGolS3* | orange1.1g007705m | F: 5’-AGCCTTGGGCGTGTTACAGA-3’  R: 5’-AGCTTCCACCACCTCTCGTG-3’ | 97 |
| *CsGolS4* | orange1.1g037463m | F: 5’-GGGCGATGGTCTTTATGCAA-3’  R: 5’-GCGACTCACCATGTCCCAAT-3’ | 91 |
| *CsGolS5* | orange1.1g041855m | F: 5’-AAAACTCGAGCAGGGATCTCG-3’  R: 5’-GTTTTCCATCCGGCAGCTCT-3’ | 88 |
| *CsGolS6* | orange1.1g024367m | F: 5’-AGCACCAAGTCTGCCAAAGC-3’  R: 5’-AGCCCTTAACCAAGCCAACG-3’ | 89 |
| *CsGolS7* | orange1.1g020230m | F: 5’-GGCGACGACAAACAGTGAAA-3’  R: 5’-CAGCAGATGGGGCATTTCTT-3’ | 95 |
| *CsGolS8* | orange1.1g019647m | F: 5’-GCCTTCTGCTGATGGTAATGC-3’  R: 5’-CACAAACTGAACGGCAGCAG-3’ | 85 |
| *GAPC2* | orange1.1g019445m | F: 5’-TCTTGCCTGCTTTGAATGGA-3’  R: 5’-TGTGAGGTCAACCACTGCGACAT-3’ | 80 |

**Table B.** Changes in metabolic abundance of *CsGolS6*-overexpressing transgenic tobacco plants. Data have been normalized by the ribitol internal standard, sample weight and the average of the relative value of control (irrigated) WT. Values are represented as means ± SE. *P* values are shown as ≤ 0.001, 0.01 and 0.05 by ***, ** and * respectively. Bold indicates a statistically significant change as compared to the control WT.

| **Metabolite** | **WT**  **Control** | **WT**  **Drought** | **L3**  **Control** | **L3**  **Drought** | **L6**  **Control** | **L6**  **Drought** | **L10**  **Control** | **L10**  **Drought** | **L12**  **Control** | **L12**  **Drought** |
| --- | --- | --- | --- | --- | --- | --- | --- | --- | --- | --- |
| **Aminoacids** | | | | | | | | | | |
| Alanine | 1 ± 0.28 | 0.49 ± 0.00 | 0.82 ± 0.18 | 0.92 ± 0.20 | 0.69 ± 0.25 | 0.35 ± 0.21 | 0.43 ± 0.24 | 0.60 ± 0.27 | 0.84 ± 0.38 | 1.89 ± 0.45 |
| Alanine_beta | 1 ± 0.13 | **1.98 ± 0.04*** | 1.12 ± 0.06 | 0.76 ± 0.22 | 0.94 ± 0.05 | 0.89 ± 0.16 | 0.64 ± 0.11 | 0.99 ± 0.27 | 0.56 ± 0.12 | 0.92 ± 0.09 |
| Asparagine | 1 ± 0.43 | 0.42 ± 0.09 | 0.14 ± 0.03 | 0.13 ± 0.06 | 0.33 ± 0.14 | 0.39 ± 0.32 | 0.15 ± 0.06 | 0.32 ± 0.16 | 0.12 ± 0.05 | 0.11 ± 0.00 |
| Aspartate | 1 ± 0.12 | 1.88 ± 0.46 | 0.88 ± 0.29 | 1.17 ± 0.32 | 2.11 ± 0.97 | 0.70 ± 0.37 | 1.48 ± 0.78 | 1.30 ± 0.26 | 0.87 ± 0.22 | 0.89 ± 0.00 |
| GABA | 1 ± 0.26 | 0.52 ± 0.03 | 0.42 ± 0.06 | 0.37 ± 0.15 | 0.62 ± 0.13 | 0.34 ± 0.06 | 0.31 ± 0.10 | 0.63 ± 0.30 | 0.27 ± 0.06 | 0.41 ± 0.04 |
| Glutamate | 1 ± 0.27 | 2.87 ± 1.16 | 1.52 ± 0.54 | **2.34 ± 0.37*** | 1.74 ± 0.68 | 1.30 ± 0.81 | 2.55 ± 0.75 | 1.85 ± 0.43 | 1.66 ± 0.13 | 1.49 ± 0.28 |
| Glutamine | 1 ± 0.38 | 0.51 ± 0.18 | 0.13 ± 0.03 | 0.16 ± 0.07 | 0.38 ± 0.14 | 0.56 ± 0.51 | 0.18 ± 0.08 | 0.45 ± 0.29 | 0.12 ± 0.03 | 0.13 ± 0.01 |
| Glycine | 1 ± 0.32 | 0.91 ± 0.16 | 0.87 ± 0.15 | 0.80 ± 0.32 | 0.58 ± 0.19 | 0.54 ± 0.15 | 0.65 ± 0.15 | 0.92 ± 0.19 | 0.45 ± 0.09 | 0.93 ± 0.08 |
| Homoserine | 1 ± 0.35 | 0.43 ± 0.13 | 1.01 ± 0.33 | 0.71 ± 0.39 | 0.56 ± 0.05 | 0.58 ± 0.27 | 0.49 ± 0.13 | 0.48 ± 0.08 | 0.28 ± 0.10 | 0.68 ± 0.09 |
| Isoleucine | 1 ± 0.33 | 1.27 ± 0.22 | 1.05 ± 0.28 | 0.95 ± 0.35 | 0.42 ± 0.13 | 0.59 ± 0.11 | 0.85 ± 0.20 | 1.16 ± 0.30 | 0.67 ± 0.14 | 0.95 ± 0.11 |
| Lysine | 1 ± 0.28 | 1.42 ± 0.26 | 1.00 ± 0.21 | 0.94 ± 0.33 | 0.41 ± 0.12 | 0.47 ± 0.04 | 0.88 ± 0.26 | 1.21 ± 0.39 | 0.73 ± 0.13 | 0.98 ± 0.08 |
| Methionine | 1 ± 0.37 | 1.28 ± 0.28 | 0.80 ± 0.25 | 0.85 ± 0.36 | 0.40 ± 0.13 | 0.42 ± 0.08 | 0.83 ± 0.21 | 1.03 ± 0.40 | 0.59 ± 0.13 | 0.79 ± 0.14 |
| Ornithine | 1 ± 0.26 | 0.89 ± 0.19 | 0.57 ± 0.15 | 0.49 ± 0.03 | 0.96 ± 0.12 | 0.51 ± 0.09 | 0.45 ± 0.05 | 0.52 ± 0.03 | 0.55 ± 0.12 | 0.51 ± 0.05 |
| Phenylalanine | 1 ± 0.41 | 1.21 ± 0.25 | 0.77 ± 0.15 | 0.68 ± 0.24 | 0.53 ± 0.19 | 0.89 ± 0.50 | 0.65 ± 0.16 | 0.91 ± 0.27 | 0.50 ± 0.09 | 0.70 ± 0.08 |
| Piroglutamato | 1 ± 0.22 | 2.23 ± 0.82 | 1.28 ± 0.38 | 1.89 ± 0.25 | 1.45 ± 0.49 | 1.17 ± 0.63 | 2.04 ± 0.53 | 1.59 ± 0.31 | 1.38 ± 0.09 | 1.26 ± 0.20 |
| Proline | 1 ± 0.32 | 1.24 ± 0.16 | 0.16 ± 0.04 | 0.30 ± 0.16 | 0.58 ± 0.31 | 0.74 ± 0.43 | 0.43 ± 0.36 | 0.58 ± 0.39 | **0.08 ± 0.02*** | 0.17 ± 0.04 |
| Serine | 1 ± 0.27 | 1.08 ± 0.30 | 0.67 ± 0.18 | 0.72 ± 0.24 | 0.60 ± 0.03 | 0.70 ± 0.37 | 0.69 ± 0.24 | 0.86 ± 0.20 | 0.44 ± 0.09 | 0.67 ± 0.01 |
| Threonine | 1 ± 0.37 | 1.00 ± 0.22 | 0.56 ± 0.13 | 0.56 ± 0.21 | 0.60 ± 0.14 | 0.56 ± 0.26 | 0.52 ± 0.16 | 0.79 ± 0.30 | 0.37 ± 0.08 | 0.54 ± 0.03 |
| Threonine_allo | 1 ± 0.37 | 1.00 ± 0.22 | 0.56 ± 0.13 | 0.56 ± 0.21 | 0.60 ±0.14 | 0.56 ± 0.26 | 0.52 ± 0.16 | 0.79 ± 0.30 | 0.37 ± 0.08 | 0.54 ± 0.03 |
| Tryptophan | 1 ± 0.26 | 2.54 ± 0.44 | 0.36 ± 0.08 | 0.73 ± 0.35 | 1.77 ± 0.45 | 1.29 ± 1.04 | 0.36 ± 0.17 | 0.80 ± 0.38 | 0.21 ± 0.11 | 0.40 ± 0.06 |
| Tyramine | 1 ± 0.10 | 0.71 ± 0.02 | **0.46 ± 0.13*** | **0.46 ± 0.14*** | 0.86 ± 0.10 | 0.64 ± 0.23 | **0.50 ± 0.06*** | **0.50 ± 0.05*** | 0.59 ± 0.11 | 0.49 ± 0.16 |
| Tyrosine | 1 ± 0.44 | 0.97 ± 0.26 | 0.53 ± 0.09 | 0.47 ± 0.16 | 0.56 ± 0.26 | 0.73 ± 0.49 | 0.46 ± 0.14 | 0.70 ± 0.28 | 0.37 ± 0.07 | 0.50 ± 0.06 |
| **Organic acids** | | | | | | | | | | |
| Ascorbate | 1 ± 0.30 | 2.01 ± 0.36 | 2.35 ± 0.43 | **3.42 ± 0.68*** | 1.16 ± 0.17 | 4.29 ± 3.01 | 7.40 ± 5.44 | 1.55 ± 0.50 | **3.31 ± 0.64*** | **2.48 ± 0.02*** |
| Acetate_ aminooxy | 1 ± 0.06 | 1.20 ± 0.05 | 0.97 ± 0.07 | 1.17 ± 0.11 | 1.14 ± 0.02 | 1.02 ± 0.13 | 0.92 ± 0.03 | 1.07 ± 0.08 | 1.07 ± 0.11 | **1.25 ± 0.01*** |
| Adipic_acid | 1 ± 0.18 | 1.03 ± 0.23 | 0.85 ± 0.04 | 0.89 ± 0.21 | 0.96 ± 0.11 | 0.71 ± 0.10 | 0.83 ± 0.11 | 0.97 ± 0.11 | 0.58 ± 0.08 | 0.79 ± 0.00 |
| Benzoate_4-hydroxy | 1 ± 0.04 | 1.10 ± 0.06 | 0.98 ± 0.02 | 1.22 ± 0.07 | 1.19 ± 0.05 | 0.86 ± 0.07 | 0.87 ± 0.02 | 1.01 ± 0.05 | 1.09 ± 0.02 | 1.17 ± 0.02 |
| Benzoate | 1 ± 0.05 | 1.05 ± 0.02 | 0.94 ± 0.04 | 0.87 ± 0.06 | 1.10 ± 0.01 | 1.09 ± 0.04 | 0.95 ± 0.04 | 0.92 ± 0.04 | **0.79 ± 0.04*** | 0.97 ± 0.02 |
| Caffeate | 1 ± 0.17 | 1.29 ± 0.16 | 1.21 ± 0.09 | 1.35 ± 0.35 | 0.89 ± 0.06 | 0.54 ± 0.10 | 0.82 ± 0.08 | 1.13 ± 0.31 | 1.31 ± 0.16 | 1.57 ± 0.01 |
| Cinamate | 1 ± 0.28 | 0.77 ± 0.24 | 1.09 ± 0.35 | 1.18 ± 0.34 | 0.75 ± 0.06 | 1.15 ± 0.23 | 0.73 ± 0.06 | 1.03 ± 0.31 | 0.46 ± 0.23 | 0.83 ± 0.13 |
| Dehydroascorbate | 1 ± 0.49 | 0.78 ± 0.13 | 1.68 ± 0.33 | 3.68 ± 1.59 | 1.19 ± 0.20 | 2.23 ± 0.72 | **2.91 ± 0.12*** | 1.19 ± 0.88 | **2.67 ± 0.34*** | 2.44 ± 0.10 |
| Dihydroxyacetone | 1 ± 0.12 | 0.91 ± 0.11 | 1.13 ± 0.18 | 1.52 ± 0.22 | 1.46 ± 0.32 | 1.15 ± 0.12 | 1.08 ± 0.07 | 1.25 ± 0.13 | 1.26 ± 0.16 | 1.36 ± 0.02 |
| Glyceraldehyde-3-phosphate | 1 ± 0.26 | 1.28 ± 0.26 | 0.72 ± 0.12 | 0.90 ± 0.42 | 0.54 ± 0.01 | 0.49 ± 0.02 | 0.51 ± 0.02 | 1.11 ± 0.42 | 0.46 ± 0.18 | 0.71 ± 0.01 |
| Glycerate | 1 ± 0.44 | 0.96 ± 0.03 | 0.33 ± 0.07 | 0.32 ± 0.09 | 1.06 ± 0.34 | 0.82 ± 0.34 | 0.51 ± 0.26 | 0.79 ± 0.41 | 0.17 ± 0.02 | 0.36 ± 0.05 |
| Galacturonate | 1 ± 0.16 | 1.08 ± 0.03 | 0.99 ± 0.16 | 0.85 ± 0.16 | 0.93 ± 0.13 | 0.67 ± 0.11 | 0.85 ± 0.18 | 0.94 ± 0.12 | 0.72 ± 0.03 | 0.86 ± 0.05 |
| Glucarate-1,4-lactone | 1 ± 0.23 | 1.07 ± 0.20 | 0.46 ± 0.13 | 0.59 ± 0.25 | 0.48 ± 0.03 | 1.12 ± 0.26 | 0.92 ± 0.27 | 1.09 ± 0.26 | 0.59 ± 0.20 | 0.23 ± 0.03 |
| Glucuronate | 1 ± 0.14 | **3.60 ± 0.09**** | 1.59 ± 0.23 | 1.64 ± 0.47 | 2.10 ± 0.31 | 1.61 ± 0.57 | 1.35 ± 0.51 | 1.57 ± 0.39 | 1.19 ± 0.24 | 1.27 ± 0.25 |
| Glucuronate-e-lactone | 1 ± 0.12 | 0.52 ± 0.06 | 0.76 ± 0.25 | 0.89 ± 0.12 | 0.81 ± 0.06 | 0.59 ± 0.12 | **0.50 ± 0.08*** | **0.55 ± 0.05*** | 1.16 ± 0.19 | 0.94 ± 0.11 |
| Maleate | 1 ± 0.03 | 0.96 ± 0.00 | 1.01 ± 0.06 | 0.99 ± 0.03 | 1.04 ± 0.02 | 1.07 ± 0.08 | 1.01 ± 0.02 | 1.03 ± 0.05 | 0.93 ± 0.02 | 1.00 ± 0.01 |
| Malonate | 1 ± 0.04 | 1.70 ± 0.21 | 1.30 ± 0.32 | 0.74 ± 0.19 | 0.75 ± 0.11 | 1.13 ± 0.15 | 0.83 ± 0.10 | 0.92 ± 0.06 | 0.88 ± 0.28 | 0.92 ± 0.15 |
| Quinate | 1 ± 0.13 | **1.71 ± 0.01*** | 1.22 ± 0.11 | 1.13 ± 0.29 | 1.02 ± 0.09 | 0.89 ± 0.18 | 1.18 ± 0.29 | 1.35 ± 0.16 | 0.99 ± 0.08 | 1.27 ± 0.04 |
| Threonate | 1 ± 0.29 | 4.39 ± 0.27 | 3.39 ± 0.85 | **2.22 ± 0.25*** | 4.41 ± 0.71 | 3.37 ± 0.83 | 3.51 ± 1.08 | **3.01 ± 0.47*** | 2.82 ± 0.83 | 2.92 ± 0.61 |
| **TCA cycle metabolites** | | | | | | | | | | |
| Aconitate | 1 ± 0.03 | 1.01 ± 0.01 | 1.12 ± 0.04 | **1.21 ± 0.06*** | 1.17 ± 0.02 | 1.05 ± 0.04 | 1.06 ± 0.02 | 1.05 ± 0.05 | **1.19 ± 0.04*** | **1.17 ± 0.00*** |
| Fumarate | 1 ± 0.39 | 0.25 ± 0.02 | 0.37 ± 0.11 | 0.19 ± 0.01 | 0.78 ± 0.29 | 0.56 ± 0.22 | 0.42 ± 0.22 | 0.50 ± 0.20 | 0.20 ± 0.07 | 0.36 ± 0.08 |
| Malate | 1 ± 0.32 | 1.12 ± 0.01 | 0.55 ± 0.10 | 0.39 ± .06 | 1.60 ± 0.11 | 1.19 ± 0.33 | 0.78 ± 0.29 | 0.89 ± 0.18 | 0.40 ± 0.04 | 0.54 ± 0.06 |
| Succinate | 1 ± 0.29 | 1.24 ± 0.22 | 0.87 ± 0.06 | 0.72 ± 0.21 | 0.99 ± 0.23 | 0.68 ± 0.22 | 0.70 ± 0.10 | 1.23 ± 0.49 | 0.61 ± 0.07 | 0.83 ± 0.04 |
| **Sugars** | | | | | | | | | | |
| Cellobiose | 1 ± 0.12 | 1.01 ± 0.17 | 0.76 ± 0.11 | 0.67 ± 0.19 | 0.87 ± 0.07 | **0.41 ± 0.03**** | **0.49 ± 0.02*** | 0.81 ± 0.18 | 0.64 ± 0.11 | 0.61 ± 0.04 |
| Erythrose | 1 ± 0.21 | 0.96 ± 0.13 | 0.67 ± 0.28 | 0.47 ± 0.17 | 0.65 ± 0.34 | 0.59 ± 0.26 | **0.30 ± 0.05*** | 0.66 ± 0.38 | 0.42 ± 0.18 | 0.79 ± 0.22 |
| Fructose | 1 ± 0.50 | 1.52 ± 0.01 | 1.63 ± 0.07 | 1.71 ± 0.07 | 1.69 ± 0.02 | 1.62 ± 0.05 | 1.62 ± 0.02 | 1.61 ± 0.07 | 1.59 ± 0.02 | 1.70 ± 0.00 |
| Fructose-6-phosphate | 1 ± 0.04 | 1.06 ± 0.05 | 1.09 ± 0.11 | 1.15 ± 0.05 | **1.12 ± 0.01*** | 1.00 ± 0.02 | 1.02 ± 0.05 | 1.15 ± 0.15 | 1.12 ± 0.10 | **1.23 ± 0.00*** |
| Fucose | 1 ± 0.06 | 1.20 ± 0.18 | 1.28 ± 0.10 | 1.17 ± 0.11 | 1.91 ± 0.61 | 0.84 ± 0.08 | 1.07 ± 0.27 | 0.96 ± 0.03 | **1.54 ± 0.08**** | 1.09 ± 0.10 |
| Galactose | 1 ± 0.04 | 0.99 ± 0.01 | 1.06 ± 0.05 | 1.11 ± 0.05 | 1.10 ± 0.01 | 1.03 ± 0.04 | 1.04 ± 0.01 | 1.04 ± 0.04 | 1.03 ± 0.01 | 1.09 ± 0.00 |
| Gentiobiose | 1 ± 0.07 | 0.92 ± 0.35 | 0.97 ± 0.29 | 1.05 ± 0.39 | **0.60 ± 0.05*** | **0.58 ± 0.12*** | 0.72 ± 0.12 | 1.44 ± 0.42 | 0.94 ± 0.16 | 0.95 ± 0.17 |
| Glucose | 1 ± 0.09 | 0.81 ± 0.03 | 0.59 ± 0.30 | 0.87 ± 0.03 | 0.89 ± 0.02 | 0.90 ± 0.07 | 0.81 ± 0.03 | 0.84 ± 0.02 | 0.88 ± 0.00 | 0.83 ± 0.01 |
| Isomaltose | 1 ± 0.20 | 0.71 ± 0.06 | 0.68 ± 0.07 | 0.65 ± 0.13 | 0.71 ± 0.04 | **0.43 ± 0.02*** | 0.51 ± 0.09 | 0.86 ± 0.22 | 0.67 ± 0.14 | 0.63 ± 0.03 |
| Kestose | 1 ± 0.09 | **0.64 ± 0.03*** | 0.65 ± 0.17 | 0.96 ± 0.40 | 0.61 ± 0.16 | 1.19 ± 0.39 | **0.47 ± 0.04**** | 0.88 ± 0.33 | **0.32 ± 0.13*** | **0.54 ± 0.01*** |
| Lactulose | 1 ± 0.22 | 0.70 ± 0.03 | 0.82 ± 0.13 | 0.80 ± 0.11 | 0.79 ± 0.08 | 0.42 ± 0.04 | 0.46 ± 0.07 | 0.78 ± 0.20 | 0.59 ± 0.09 | 0.64 ± 0.05 |
| Maltose | 1 ± 0.08 | 0.90 ± 0.04 | **1.75 ± 0.24*** | 1.35 ± 0.20 | 0.96 ± 0.14 | 0.99 ± 0.17 | 1.09 ± 0.25 | 1.11 ± 0.11 | 1.26 ± 0.11 | 1.27 ± 0.06 |
| Maltotriose | 1 ± 0.19 | 0.62 ± 0.17 | 1.64 ± 0.46 | 0.87 ± 0.21 | 1.09 ± 0.16 | 0.65 ± 0.08 | 0.89 ± 0.20 | 0.93 ± 0.09 | 0.81 ± 0.21 | 1.15 ± 0.11 |
| Mannose | 1 ± 0.04 | 0.99 ± 0.01 | 1.06 ± 0.05 | 1.11 ± 0.05 | 1.10 ± 0.01 | 1.03 ± 0.04 | 1.04 ± 0.01 | 1.04 ± 0.04 | 1.03 ± 0.01 | 1.09 ± 0.00 |
| Melibiose | 1 ± 0.19 | 0.90 ± 0.18 | 0.72 ± 0.09 | 1.06 ± 0.39 | **0.29 ± 0.10*** | 0.45 ± 0.09 | 0.62 ± 0.06 | 1.54 ± 0.41 | 0.75 ± 0.16 | 0.71 ± 0.07 |
| Palatinose | 1 ± 0.15 | 0.93 ± 0.13 | 1.06 ± 0.16 | 1.07 ± 0.37 | 0.58 ± 0.05 | **0.50 ± 0.06*** | 0.74 ± 0.13 | 1.27 ± 0.27 | 1.13 ± 0.14 | 1.01 ± 0.00 |
| Raffinose | 1 ± 0.09 | **0.64 ± 0.03*** | 0.65 ± 0.17 | 0.96 ± 0.40 | 0.61 ± 0.16 | 1.19 ± 0.39 | **0.47 ± 0.04**** | 0.88 ± 0.33 | **0.32 ± 0.13*** | **0.54 ± 0.01*** |
| Ribose-5-phosphate | 1 ± 0.02 | 3.22 ± 0.33 | 1.12 ± 0.10 | 1.60 ± 0.27 | 1.32 ± 0.01 | 0.98 ± 0.50 | 1.49 ± 0.47 | 1.17 ± 0.13 | **1.84 ± 0.14**** | **1.77 ± 0.17*** |
| Sorbose | 1 ± 0.03 | 0.98 ± 0.01 | 1.04 ± 0.05 | 1.09 ± 0.04 | 1.08 ± 0.00 | 1.02 ± 0.04 | 1.03 ± 0.01 | 1.03 ± 0.03 | 1.02 ± 0.01 | 1.06 ± 0.00 |
| Sucrose | 1 ± 0.06 | 1.01 ± 0.01 | 0.81 ± 0.22 | 1.00 ± 0.11 | 1.16 ± 0.01 | 1.09 ± 0.05 | 0.82 ± 0.14 | 0.93 ± 0.08 | 0.68 ± 0.20 | 0.71 ± 0.24 |
| Trehalose | 1 ± 0.20 | 0.87 ± 0.14 | 0.77 ± 0.15 | 0.87 ± 0.21 | 0.67 ± 0.05 | 0.58 ± 0.02 | 0.56 ± 0.06 | 1.01 ± 0.18 | 0.85 ± 0.07 | 0.72 ± 0.03 |
| Turanose | 1 ± 0.15 | 0.71 ± 0.06 | 1.02 ± 0.28 | 0.85 ± 0.18 | 1.00 ± 0.01 | 0.76 ± 0.15 | 0.64 ± 0.16 | 0.88 ± 0.17 | 0.80 ± 0.03 | 0.90 ± 0.06 |
| Xylose | 1 ± 0.13 | 1.31 ± 0.00 | 1.39 ± 0.32 | 1.12 ± 0.05 | 2.14 ± 0.61 | 0.96 ± 0.10 | 0.99 ± 0.34 | 0.96 ± 0.07 | **1.75 ± 0.23*** | 1.10 ± 0.04 |
| **Sugar alcohol** | | | | | | | | | | |
| allo_Inositol | 1 ± 0.32 | 0.61 ± 0.13 | 0.43 ± 0.04 | 0.45 ± 0.03 | 0.44 ± 0.05 | 0.51 ± 0.18 | 0.61 ± 0.18 | 0.80 ± 0.22 | 0.42 ± 0.06 | 0.36 ± 0.01 |
| Galactinol | 1 ± 0.16 | 1.34 ± 0.07 | 1.15 ± 0.28 | 1.58 ± 0.47 | 0.93 ± 0.21 | 1.39 ± 0.40 | 0.85 ± 0.09 | 1.16 ± 0.31 | 1.05 ± 0.32 | 0.99 ± 0.13 |
| Maltitol | 1 ± 0.02 | 0.72 ± 0.42 | 1.22 ± 0.28 | 1.23 ± 0.32 | **0.33 ± 0.19*** | 0.72 ± 0.12 | 0.95 ± 0.20 | 1.30 ± 0.19 | 1.31 ± 0.15 | **1.23 ± 0.00**** |
| myo-Inositol | 1 ± 0.04 | 1.00 ± 0.00 | 1.07 ± 0.05 | 1.12 ± 0.02 | 1.08 ± 0.01 | 1.39 ± 0.39 | 1.06 ± 0.01 | 1.05 ± 0.04 | 1.03 ± 0.02 | 1.07 ± 0.01 |
| **Secondary metabolism** | | | | | | | | | | |
| alfa-Tocopherol | 1 ± 0.35 | 1.87 ± 0.62 | **2.49 ± 0.30*** | 1.73 ± 0.59 | 1.32 ± 0.62 | 1.08 ± 0.43 | 2.38 ± 0.93 | 1.11 ± 0.12 | **3.63 ± 0.70*** | 2.83 ± 0.52 |
| beta-Tocopherol | 1 ± 0.19 | 1.23 ± 0.01 | 1.32 ± 0.05 | 1.36 ± 0.04 | 1.05 ± 0.21 | 1.29 ± 0.02 | 1.32 ± 0.03 | 1.28 ± 0.06 | 1.37 ± 0.08 | 2.02 ± 0.40 |
| Dopamine | 1 ± 0.18 | 0.43 ± 0.07 | **0.27 ± 0.10*** | **0.24 ± 0.08*** | **0.43 ± 0.00*** | **0.33 ± 0.16*** | **0.26 ± 0.09*** | **0.29 ± 0.08*** | **0.27 ± 0.01*** | **0.32 ± 0.09*** |
| Nicotinate | 1 ± 0.08 | 0.98 ± 0.13 | 0.79 ± 0.04 | 0.74 ± 0.10 | 0.84 ± 0.08 | 0.98 ± 0.07 | 0.92 ± 0.06 | 0.95 ± 0.07 | 0.81 ± 0.01 | **0.67 ± 0.00*** |
| Nicotinate_6-hidroxi | 1 ± 0.01 | 1.04 ± 0.05 | 1.17 ± 0.07 | **1.51 ± 0.11**** | 1.70 ± 0.01 | **0.28 ± 0.10**** | **0.64 ± 0.05**** | 0.85 ± 0.09 | **1.58 ± 0.01***** | **1.64 ± 0.03***** |
| Quinate-3-caffeoyl | 1 ± 0.36 | 1.83 ± 0.62 | 2.31 ± 0.37 | 1.71 ± 0.56 | 1.26 ± 0.61 | 1.05 ± 0.41 | 2.33 ± 0.87 | 1.06 ± 0.09 | **3.59 ± 0.65*** | 2.76 ± 0.51 |
| Salicylate | 1 ± 0.10 | 4.62 ± 2.16 | 0.93 ± 0.06 | 0.96 ± 0.13 | 2.51 ± 0.80 | 0.75 ± 0.21 | 0.68 ± 0.06 | 0.80 ± 0.06 | 0.93 ± 0.02 | 1.01 ± 0.04 |
| Shikimate | 1 ± 0.03 | 1.02 ± 0.01 | 1.09 ± 0.05 | 1.14 ± 0.04 | 1.13 ± 0.00 | 1.02 ± 0.04 | 1.03 ± 0.01 | 1.04 ± 0.04 | 1.07 ± 0.02 | 1.09 ± 0.01 |
| **Polyamines** | | | | | | | | | | |
| Spermidine | 1 ± 0.08 | 3.02 ± 0.67 | **1.91 ± 0.17*** | **2.64 ± 0.58*** | 1.99 ± 0.77 | 0.98 ± 0.08 | **2.07 ± 0.25*** | **1.73 ± 0.14*** | **2.26 ± 0.21**** | **1.56 ± 0.00*** |
| Putrescine | 1 ± 0.30 | 0.32 ± 0.13 | **0.15 ± 0.07*** | **0.13 ± 0.03**** | 0.81 ± 0.39 | 0.62 ± 0.48 | **0.07 ± 0.07*** | 0.35 ± 0.17 | **0.13 ± 0.05**** | **0.23 ± 0.01*** |
| **Others** | | | | | | | | | | |
| Adenine | 1 ± 0.14 | 0.96 ± 0.20 | 1.17 ± 0.40 | 1.91 ± 0.64 | 1.35 ± 0.15 | 1.05 ± 0.12 | 1.28 ±0.41 | 1.22 ± 0.40 | 1.87 ± 0.49 | 1.19 ± 0.11 |
| Calystegine B2 | 1 ± 0.08 | 1.25 ± 0.10 | 1.55 ± 0.31 | 1.23 ± 0.17 | 1.74 ± 0.45 | 0.87 ± 0.12 | 1.54 ± 0.54 | 1.18 ± 0.14 | **2.68 ± 0.59*** | 1.60 ± 0.20 |
| Cystathionine | 1 ± 0.07 | 1.51 ± 0.12 | 0.94 ± 0.13 | 1.08 ± 0.16 | 1.42 ± 0.11 | 1.01 ± 0.18 | 0.89 ± 0.07 | 1.11 ± 0.18 | 1.10 ± 0.06 | 0.98 ± 0.12 |
| Glucoheptonate | 1 ± 0.11 | 1.51 ± 0.04 | 0.98 ± 0.24 | 1.15 ± 0.17 | 1.94 ± 0.41 | 1.29 ± 0.36 | 0.97 ± 0.14 | 1.22 ± 0.13 | 1.54 ± 0.31 | 1.00 ± 0.22 |
| Glucoheptose | 1 ± 0.05 | 1.20 ± 0.00 | 1.13 ± 0.14 | 1.58 ± 0.34 | 1.28 ± 0.07 | 1.13 ± 0.09 | **1.22 ± 0.03*** | 1.29 ± 0.21 | **1.33 ± 0.10*** | 1.14 ± 0.07 |
| Glucosone | 1 ± 0.35 | 1.48 ± 0.03 | 0.46 ± 0.11 | 0.85 ± 0.39 | 0.61 ± 0.12 | 0.50 ± 0.18 | 0.56 ± 0.23 | 0.94 ± 0.44 | 0.47 ± 0.18 | 0.42 ± 0.12 |
| Gluconate-1,5-lactone | 1 ± 0.04 | 1.22 ± 0.00 | 1.18 ± 0.18 | **1.26 ± 0.04*** | 1.30 ± 0.12 | 1.02 ± 0.10 | 1.04 ± 0.12 | 1.09 ± 0.08 | 1.25 ± 0.09 | 1.14 ± 0.02 |
| Glucopyranoside | 1 ± 0.06 | **1.94 ± 0.00***** | **1.45 ± 0.14*** | **1.69 ± 0.23*** | 1.64 ± 0.04 | 1.45 ± 0.33 | 1.28 ± 0.17 | 1.57 ± 0.23 | **1.71 ± 0.11**** | 1.47 ± 0.19 |
| Pantothenate | 1 ± 0.08 | 0.93 ± 0.14 | 1.20 ± 0.38 | 1.84 ± 0.66 | 1.38 ± 0.17 | 1.08 ± 0.14 | 1.29 ± 0.40 | 1.22 ± 0.39 | 1.88 ± 0.41 | 1.13 ± 0.08 |
| Pyridoxine | 1 ± 0.10 | 1.12 ± 0.05 | 0.94 ± 0.05 | 1.18 ± 0.10 | 1.17 ± 0.05 | 0.66 ± 0.11 | **0.67 ± 0.03*** | 0.77 ± 0.00 | 0.73 ± 0.03 | 0.65 ± 0.03 |
| Saccharopine | 1 ± 0.43 | 0.16 ± 0.07 | 0.02 ± 0.01 | 0.01 ± 0.01 | 0.43 ± 0.24 | 0.14 ± 0.12 | 0.01 ± 0.00 | 0.22 ± 0.18 | 0.01 ± 0.01 | 0.01 ± 0.00 |
| Uracil | 1 ± 0.14 | 0.85 ± 0.04 | 0.83 ± 0.02 | 0.80 ± 0.08 | 0.91 ± 0.11 | 0.66 ± 0.06 | **0.55 ± 0.00*** | 0.69 ± 0.10 | 0.62 ± 0.02 | 0.69 ± 0.01 |
| Xanthosine | 1 ± 0.50 | 4.10 ± 0.05 | 1.16 ± 0.67 | 2.23 ± 1.43 | 1.61 ± 0.04 | 2.55 ± 1.46 | 1.55 ± 0.85 | 2.01 ± 1.29 | 1.51 ± 0.72 | 0.52 ± 0.26 |

CsGolS7 ---------------------MSFVEITEPIMNVPKRAYVTFLAGNGDYVKGVVGLAKGL 39

CsGolS6 ---------MAPDIT---PTTITKTTSLSKAPSLPKRAYVTFLAGDGDYWKGVVGLVKGL 48

CsGolS8 --------MAPPELV---QTAVKPAGLGAKPASLPGRAYVTFLAGNGDYVKGVVGLAKGL 49

CsGolS1 QLPVGSCELALPLRDKDR----------VYSGSVHREAYATILHSAHVYVCGAIAAAQSI 328

CsGolS2 LLPVGSCELAVPLKAKEN----------FYSERARREAYATILHSAHVYVCGAIAAAQSI 212

CsGolS3 SLPVGSCKLALPLWGQGVDEVYDLSRIKSATKIAKREAYATVLHSSEAYVCGAITLAQSL 320

CsGolS4 ------------------------------------------------------------ 114

CsGolS5 ------------------------------------------------------------ 84

CsGolS7 RKAKSEYPLVVAILPDVPEDHRQILESQGCIVREIEPVYPPENQTEFAMAYYVINYSKLR 99

CsGolS6 RKAKSKYPLLVAMLPDVPEEHRKILIEQGCILREIEPVYPPENQTQFAMAYYVINYSKLR 108

CsGolS8 RKVKTAYPLVVAVLPDVPEEHRNILESQGCIVREIEPVYPPDNQTQYAMAYYVINYSKLR 109

CsGolS1 RMSGSTRDLVILVDETISAYHRSGLEAAGWKVRTIQRIRNPKAEK---DAYNEWNYSKFR 385

CsGolS2 RMAGSTRDLVILVDETISDYHRGGLEAAGWKIHTIQRIRNPKAER---DAYNEWNYSKFR 269

CsGolS3 RKTGTKRDLVLLIDNSISIPKREALTAAGWKIRIIKRIRNPRAEK---KTYNEYNYSKFR 377

CsGolS4 ----------------------------------AKLLLLLLKQK---DSYNEWNYSKLR 137

CsGolS5 ----------------------------------R--IRSPFAKK---DSYNEWNYSKLR 105

CsGolS7 IWEFVEYEKMIYLDGDIQVFDNIDHLFDAPDGYFYAVMDCFCEKTWSNSPQFTIGYCQQC 159

CsGolS6 IWEFVEYSKMIYLDGDIQVFDNIDHLFDLPDGYFYAVMDCFCEKTWSHTPQFKIGYCQQC 168

CsGolS8 IWEFVEYSKMIYLDGDIQVFENIDHLFDLPDGYFYAVMDCFCEKTWSKTPQYKIGYCQQC 169

CsGolS1 LWQLTDYDKIIFIDADLLILRNIDFLFGMPEIS--------------------------- 418

CsGolS2 LWQLTDYDKIIFIDADLLILRNIDFLFEMPEIT--------------------------- 302

CsGolS3 LWQLTDYDKIIFIDADIIVLRNLDLLFHFPQMS--------------------------- 410

CsGolS4 VWQLIEYDKIIFIDSDLLVLKNIDEFFFYPEFS--------------------------- 170

CsGolS5 -------------------------FFFYPELS--------------------------- 113

CsGolS7 PEKVQWPVEMGSPPPLYFNAGMFVYEPNLLTYHDLLETVKVTPPTIFAEQDFLNMYFKDI 219

CsGolS6 PDKVKWPAELGPKPALYFNAGMFVFEPSLSTYHDLLETVQITTPTSFAEQDFLNMYFRDI 228

CsGolS8 PDRVRWPAEMGEPPALYFNAGMFVFEPSISTYHDLLETVKVTPPTTFAEQDFLNMYFKHI 229

CsGolS1 ---------ATGNNGTMFNSGVMVIEPSSCTFQLLMDHINEFESYNGGDQGYLNEVFTW- 468

CsGolS2 ---------ATGNNATLFNSGVMVVEPSNCTFQLLMDHIYEIESYNGGDQGYLNEIFTW- 352

CsGolS3 ---------ATGNDIWIFNSGIMVIEPSNCTFRILMSKRKEIVSYNGGDQGFLNEVYVW- 460

CsGolS4 ---------AAGTNKVLFNSGVMVIEPSLCKFEDLTLKSFKVSSYNGGDQGFLNEVFTW- 220

CsGolS5 ---------AAGNDKVLFNSGVMVIEPSLCKFEDLMLKSFQVSSYNGGDQGFLNEVFTW- 163

CsGolS7 YKPIPPTYNLVVAMLWRHLEN-----------VDVDKVKVVHYCAAGSKPWRFTGKE--- 265

CsGolS6 YRPIPPIYNLVVAMLWRHPEN-----------VEADKAKVVHYCAAVSTIL--------- 268

CsGolS8 YKPIPLVYNLVLAMLWRHPEN-----------VELDKVKVVHYCAAGSKPWRFTGEE--- 275

CsGolS1 WHRIPKHMNFLK-HFWFGDEEEVKQKKTRLFGADPPILYVLHY--LGMKPWLCFRDYDCN 525

CsGolS2 WHRIPKHMNFLK-HFWEGDEEEKKHMKIRLFGADPPILYVLHY--LGNKPWLCFRDYDCN 409

CsGolS3 WHRLPRRVNFLK-NFWANTTLEA-SVKNHLFGADPPKLYAIHY--LGLKPWACYRDYDCN 516

CsGolS4 WHRLPKRINHLK-VFSKQDDKEH-QV--------GDGLYAIHY--LGLKPWMCYKDYDCN 268

CsGolS5 WHRLPKRINHLK-VFSKQDDKEH-QV--------GDGLYAIHY--LGLKPWM-------- 203

**Fig A.** Alignment of CsGolS conserved glycosyltransferase family 8 (GT8) domain, indicated by a black line above the amino acids. Box indicates putative manganese-binding motif (DXD).


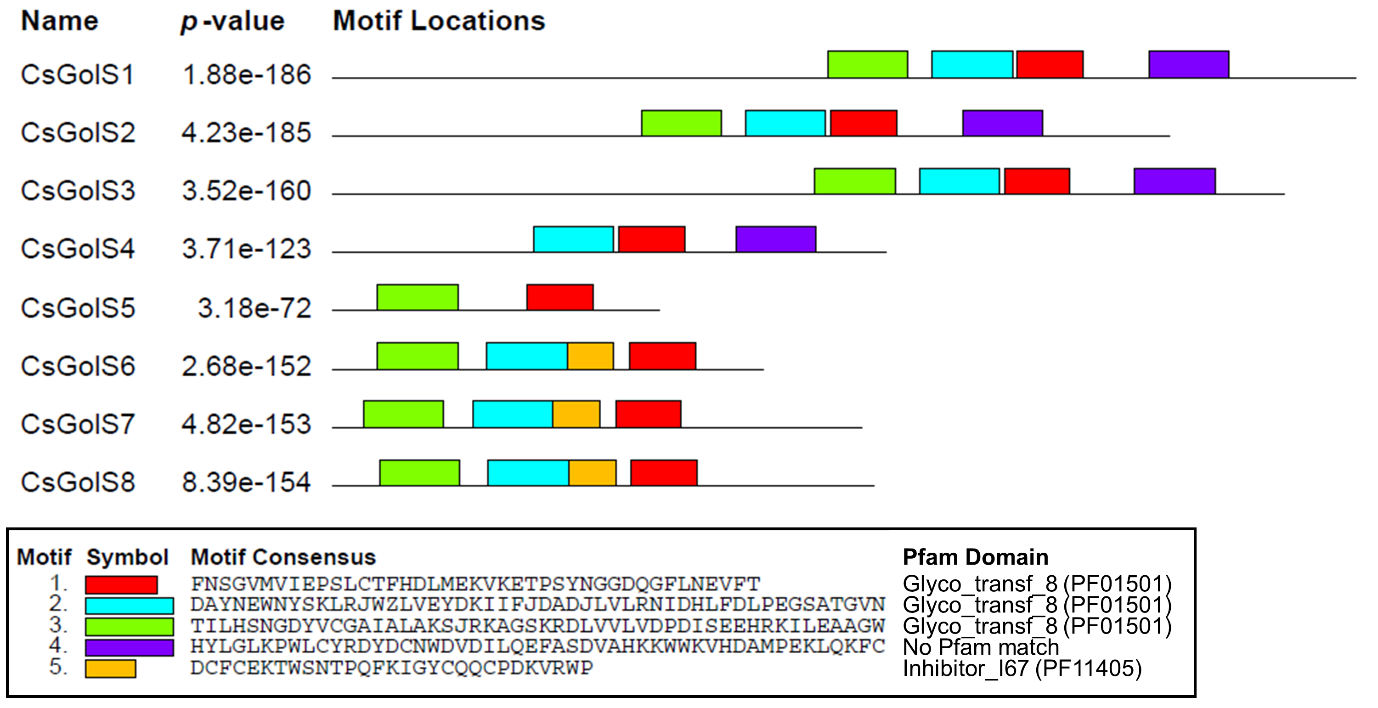


**Fig B.** Block diagram of conserved motifs in CsGolS proteins. Each motif was represented with a box in different color: motif 1, red; motif 2, cyan; motif 3, green; motif 4, blue; and motif 5, yellow.


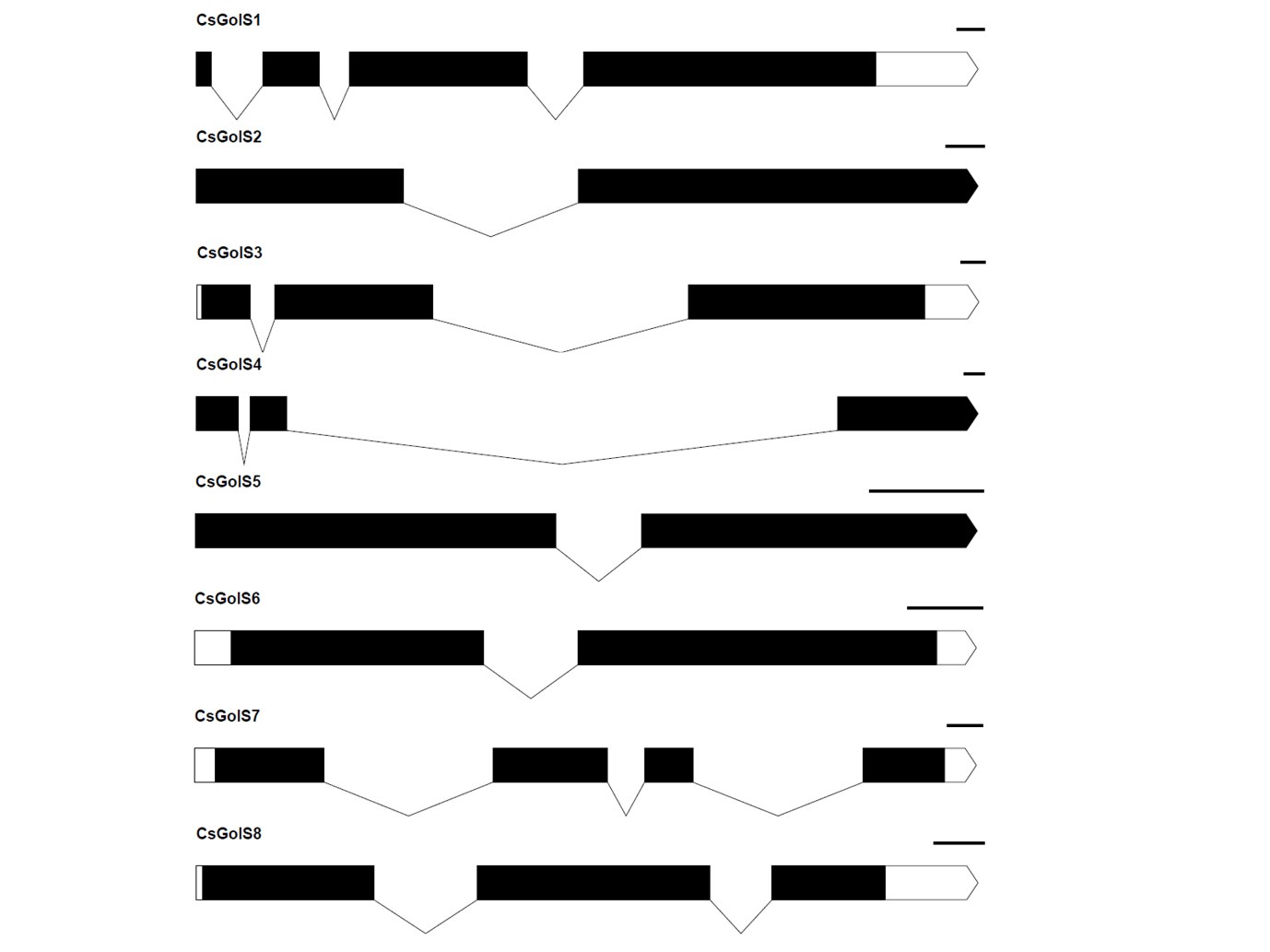


**Fig C.** Analysis of exon-intron structures of the sweet orange *GolS* genes. Open boxes correspond to 5′ and 3′ untranslated regions (UTR) and exons and introns are represented by filled boxes and black lines, respectively. Reference scale bar of 100 bp.


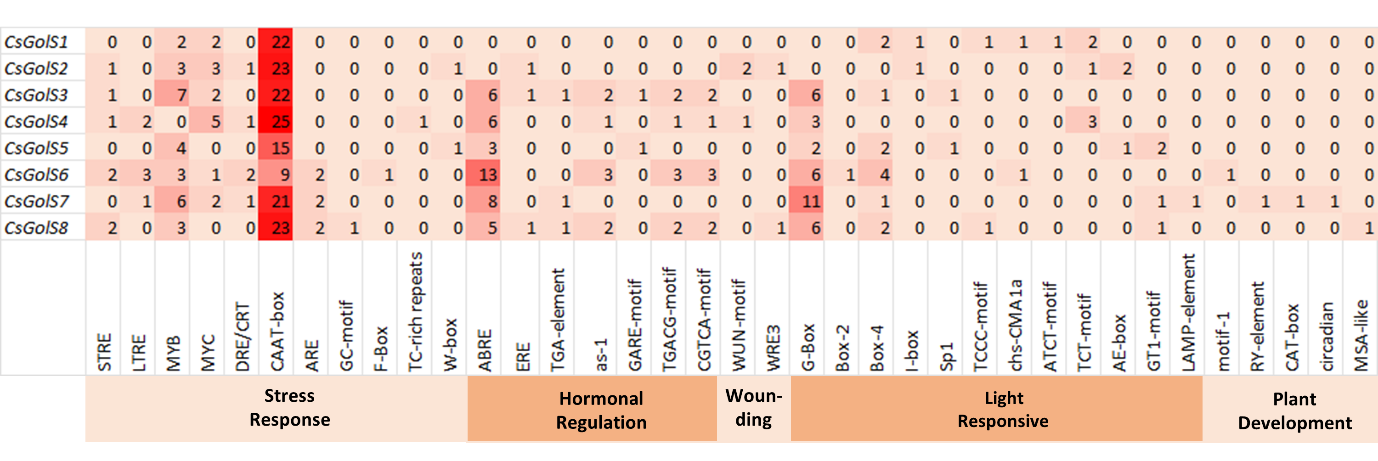


**Fig D.** Predicted *cis*-acting regulatory elements, and their respective numbers, in the promoter regions of sweet orange *GolS* genes.


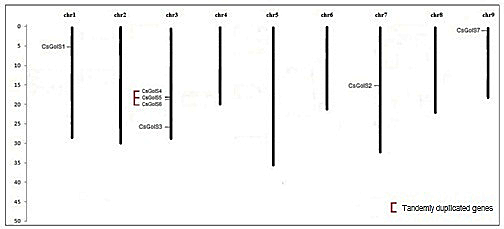


**Fig E.** Chromosomal locations of *CsGolS*. The scale in Mb.


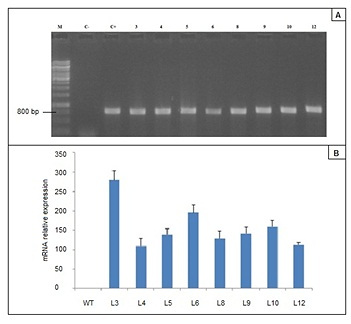


Fig F. Overexpression of *CsGolS6* in transgenic tobacco plants. (A) PCR amplification of *nptII* gene in genomic DNA from leaves of WT and transgenic tobacco plants. (B) qPCR analysis of *CsGolS6* expression in transgenic tobacco plants.


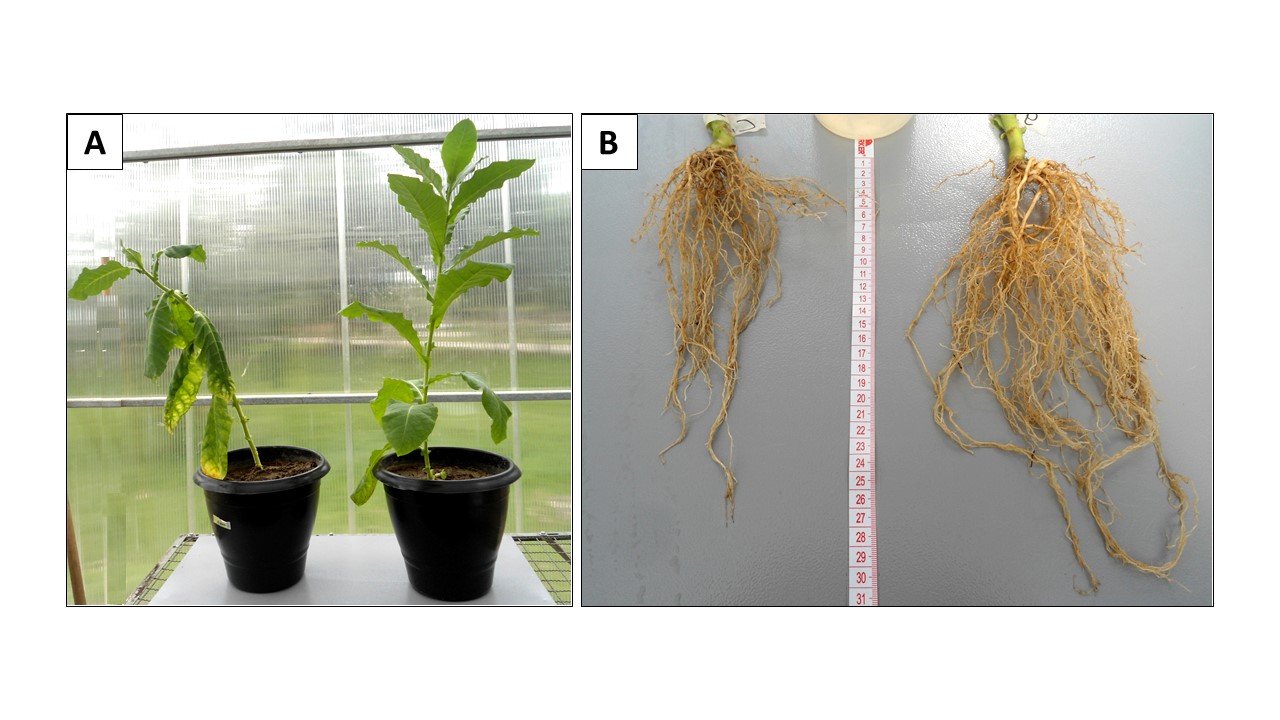


Fig G. Phenotype of drought-stressed WT (left) and *CsGolS6*-overexpressing transgenic (right) lines. Pictures of the aerial part (A) and roots (B) were taken 45 days after the beginning of drought stress experiment. See materials and methods for further details.
